# Glycosyltransferase Family 61 in Liliopsida (Monocot): The Story of a Gene Family Expansion

**DOI:** 10.3389/fpls.2018.01843

**Published:** 2018-12-11

**Authors:** Alberto Cenci, Nathalie Chantret, Mathieu Rouard

**Affiliations:** ^1^Bioversity International, Parc Scientifique Agropolis II, Montpellier, France; ^2^AGAP, INRA, CIRAD, Université de Montpellier, Montpellier, France

**Keywords:** glycosyltransferase family 61, Liliopsida, gene family expansion, positive selection footprints, orthologous genes, phylogeny

## Abstract

Plant cell walls play a fundamental role in several plant traits and also influence crop use as livestock nutrition or biofuel production. The Glycosyltransferase family 61 (GT61) is involved in the synthesis of cell wall xylans. In grasses (Poaceae), a copy number expansion was reported for the GT61 family, and raised the question of the evolutionary history of this gene family in a broader taxonomic context. A phylogenetic study was performed on GT61 members from 13 species representing the major angiosperm clades, in order to classify the genes, reconstruct the evolutionary history of this gene family and study its expansion in monocots. Four orthogroups (OG) were identified in angiosperms with two of them displaying a copy number expansion in monocots. These copy number expansions resulted from both tandem and segmental duplications during the genome evolution of monocot lineages. Positive selection footprints were detected on the ancestral branch leading to one of the orthogroups suggesting that the gene number expansion was accompanied by functional diversification, at least partially. We propose an OG-based classification framework for the GT61 genes at different taxonomic levels of the angiosperm useful for any further functional or translational biology study.

## Introduction

Glycosyltransferases (GTs) constitute a large superfamily of enzymes that catalyze the assembling of monosaccharide moieties into linear and branched glycan chains (Rini et al., 2009). GTs have been subdivided into several families (Coutinho et al., 2003) with 105 of them identified up to now (Lombard et al., 2014). Among them, the glycosyltransferase family 61 (GT61) contains genes strongly believed to play a central role in the synthesis and feruloylation of Arabinoxylan, the major components of cell walls in grasses (Mitchell et al., 2007). These components are known to play a crucial role against pathogen penetration, manufacturing processes for human and animal consumption as well as alcohol and biofuel production (Freeman et al., 2017).

The GT61 proteins are characterized by the presence of a conserved domain of unknown function (Pfam DUF563) in the C-terminal portion and by a N-terminal putative transmembrane domain (Chiniquy et al., 2012). Until now, only four gene have been functionally characterized: the *Arabidopsis thaliana* XylT (β1,2-xylosyltransferase) (Strasser et al., 2000), the *Oryza sativa* Xax1 (Xylosyltransferase) (Chiniquy et al., 2012) and the *Triticum aestivum* TaXAT1 and TaXAT2 (xylan arabinosyltransferases) (Anders et al., 2012). Additionally, some other GT61 genes members has been shown to be involved in the synthesis of Xyl-Rich mucilage polymers in *A. thaliana* (Muci21) (Voiniciuc et al., 2015) and in *Plantago* subspecies (Phan et al., 2016).

**FIGURE 1:**
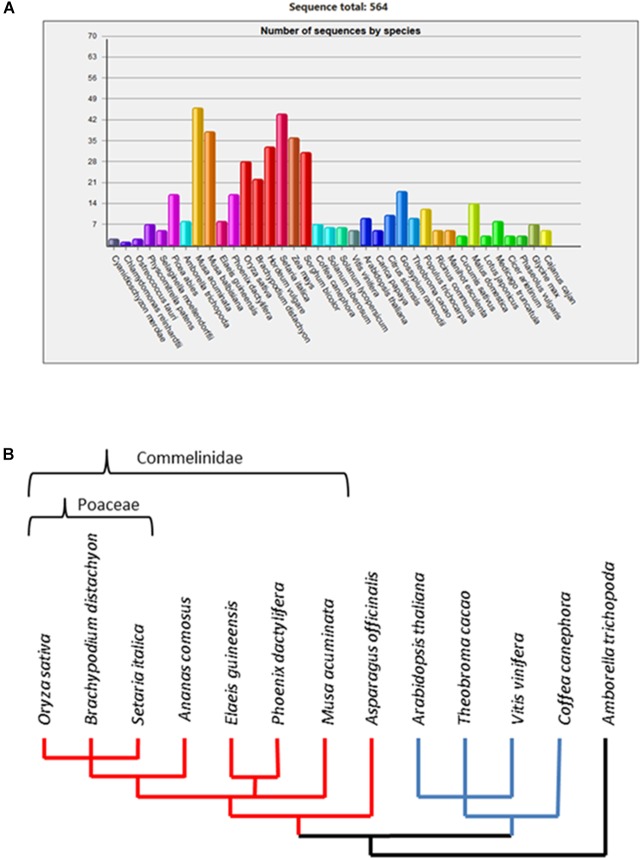
**(A)** Number of GT61 family members annotated in 37 plant genomes from the GreenPhyl database. Monocot species span from *Musa acuminata* to *Sorghum bicolor*; dicot species span from *Coffea canephora* to *Cajanus cajan*. **(B)** Dendrogram of phylogenetic relationships among the studied species.

Previous studies (Anders et al., 2012; Voiniciuc et al., 2015) consistently divided the GT61 genes family in three major clades, named A, B, and C. Clade C is the most differentiated and contains usually one gene per species whereas clades A and B contain several members per species. In clade A, a gene expansion was shown in the Poaceae family based on sequences from *O. sativa* and *Sorghum bicolor*. However, comparative genomics databases based on a wider plant genome sampling such as GreenPhyl (Rouard et al., 2010) indicate possible gene copy amplification in other monocots beyond the Poaceae (Figure 1A). Gene family expansions can be due to both small size duplications (tandem duplications) and large scale duplications [segmental or whole genome duplications (WGDs)]. In the first case, duplicated copies of the genes remain physically close in the genome (forming gene clusters) while for large scale duplications, the genes are more distant with reduced physical interactions between them. Whatever the duplication mode, each copy accumulates independent mutations (genic conversion apart) and the classically described evolutionary fates of duplicated genes are either the loss of redundant copies, subfunctionalization (maintenance of both copies to assure the original function) or neofunctionalization (acquisition of a new function retained over time by natural selection due to a selective effect increasing fitness) [Moore and Purugganan (2005) and Innan and Kondrashov (2010)]. Somehow, the persistence of two or more copies of a gene over the course of evolution is a clue of functional differentiation among the copies (Fischer et al., 2014). Such a gene expansion and selective pressure of the GT61 gene duplicates have not been examined yet.

The objectives of this study are first to investigate the homology relationships in the GT61 by identifying orthogroups (OG) and thus providing an OG-based classification framework for this family (Cenci et al., 2014; Cenci and Rouard, 2017). Orthogroups are defined as group of genes of a given sample of species that descended from a single ancestral gene. Since the functional annotation transfer relies on the accurate identification of orthologous sequences between genomes, this work will likely be useful for any further classification and gene function assignment in the GT61 gene family. The second objective was to identify the type of duplication events that occurred in the GT61 beyond Poales in the monocots and clarify whether patterns of functional differentiation were present.

Cell walls in flowering plants (angiosperms) differ in their characteristics based on the presence of a network of either Xyloglucans (XyGs) present in dicots and non-commelinids monocots (e.g., Asparagales) or Galacturonoarabinoxylans (GAXs) specific to monocots commelinids (e.g., Arecales, Zingiberales, and Poales) (Vogel, 2008). Consequently, we analyzed the whole set of GT61 sequences in a broad taxonomic panel composed of a basal monocot (*Asparagus officinalis*), the main clades of the monocot commelinids (*Musa acuminata* for Zingiberales, *Phoenix dactylifera* and *Elaeis guineensis* for Arecales, *Ananas comosus* for basal Poales and *Setaria italica*, *Brachypodium distachyon*, and *O. sativa* for Poaceae), dicots (*Coffea canephora* for asterids, *A. thaliana*, *Vitis vinifera*, and *Theobroma cacao* for rosids) and the basal angiosperm *Amborella trichopoda* as outgroup for both monocot and dicots (Figure 1B).

## Materials and Methods

### Sequence Identification and Conserved Motif Analysis of GT61 Genes

GT61 protein sequences from *M. acuminata* were retrieved from GreenPhyl database (Rouard et al., 2010) and used to search GT61 sequences of other species with BLASTp (score > 200) in their respective NCBI Annotation Release from *A. trichopoda* (release 101, species code AMBTC), *V. vinifera* (102, VITVI), *T. cacao* (100, THECC), *P. dactylifera* (101, PHODA), *E. guineensis* (101, ELAGV), *S. italica* (103, SETIT), *B. distachyon* (103, BRADI), *O. sativa* (102, ORYSA), and *A. officinalis* (100, ASPOF) or from species specific sequence databases: *A. thaliana* (ARATH) from TAIR10.1 (Berardini et al., 2015), *M. acuminata* (MUSAC) v2, from the Banana Genome Hub (Droc et al., 2013), *C. canephora* (COFCA), from the Coffee Genome Hub (Dereeper et al., 2015) and *A. comosus* (ANACO) from Plaza v4 (Van Bel et al., 2018). Sequences were manually curated to verify their gene structure and when necessary exon introns boundaries were corrected. A list detailing species and annotation for all the sequences used as well as a FASTA file containing them are available in Supplementary Datas S1, S2. A five-digit species code at the end of sequence names (as reported above between brackets) indicates the relative species. GT61 genes’ physical locations along the genome were determined for all the species. When chromosome pseudomolecules were unavailable, the assignation was based on scaffold coordinates. GT61 genes separated by no more than five other genes were considered in tandem cluster.

### Alignment, Phylogenetic Analysis and Orthogroup Identification Method

Phylogenetic analyses were performed on protein sequence alignments obtained with the MAFFT program (Katoh and Standley, 2013) via the EMBL-EBI bioinformatics interface (Li et al., 2015) using default parameters. Conserved blocks were extracted from the alignments with Gblocks (Castresana, 2000). The selection of conserved blocks was performed by allowing: (i) smaller final blocks, (ii) gap positions within the final blocks, and (iii) less strict flanking positions. Phylogenetic trees were built with PhyML (Guindon and Gascuel, 2003) available at phylogeny.fr (Dereeper et al., 2008) using an LG substitution model and the Approximate Likelihood-Ratio Test (aLRT) as statistical tests for branch support (Guindon et al., 2009). Phylogenetic trees were visualized with MEGA6 (Tamura et al., 2013).

In this study, orthogroups (OG) were visually delineated with regards to the angiosperm species tree in Figure 1B (source NCBI taxonomy). OGs were thus identified based on gene trees as the clades including both monocot and dicot, implying the existence of a common ancestor gene before the monocot/dicot lineage split. The same method was applied for GT61 genes that underwent additional copy amplification in monocot lineage with commelinids and Poaceae divergence as reference taxonomic level.

### PAML Analysis

In order to investigate the selection pressures driving evolution of the GT61 family, different models allowing the dN/dS ratio (ω, i.e., the non-synonymous on synonymous substitution rate ratio) to vary according to branches, sites or both, were tested using the codeml program of the PAML4 software (Yang, 1997). Three kinds of models were used: ‘site’ models, wherein the dN/dS ratio is allowed to vary between sites; ‘branch’ models wherein the dN/dS ratio is allowed to vary between branches; and ‘branch-site’ models wherein the dN/dS ratio is allowed to vary between both branches and sites. Site models were implemented in homemade python scripts, relying on the egglib package (De Mita and Siol, 2012).

The ‘site’ models were used to test whether positive selection drove the differentiation between paralogous sequences within each species, as performed in Fischer et al. (2016). Two models were tested: the nearly neutral model (M8a) assumes that codons evolve either neutrally or under purifying selection whereas the positive selection model (M8) assumes positive selection acting on certain codons. Likelihood ratio tests (LRTs) were performed to compare M8 with M8a and, hence, to detect sequences groups (species) for which models that include positive selection are more likely to occur than models that do not. When models with positive selection were more likely, Bayes empirical method was used to calculate the posterior probabilities at each codon and to detect those under positive selection (i.e., those with a posterior probability of having a dN/dS > 1 above 95%). Sites detected to be under positive selection at the codon level were manually validated according to alignment quality and reliability.

To determine if each of the six branches leading to subgroups A1 to A6 underwent a significant different selective pressure compared to all the other branches of the tree, the ‘branch’ and ‘branch-site’ models were used. Each of the six branches was tested individually by comparing the likelihood of a ‘branch’ model allowing the dN/dS to take a different value for the tested branch with the likelihood of a ‘null’ model. The null model was defined as follow. Two different values of dN/dS are allowed: a first value of dN/dS on the ‘outgroup’ branches (G group) and another value of dN/dS on all the other branches of the tree. As multiple testing is implicit in this method, the *p*-values were corrected using the total number of branch partitions tested (i.e., 6).

Branch partitions tested with ‘branch-site’ models were the same as for the ‘branch’ models. Two models were compared: the null model (A0), in which sites on all branches (including the G group) evolved under the same selective pressure (purifying or neutral), was compared to a model including positive selection (model A) in which some sites on the tested branch evolved under positive selection, whereas sites on the rest of branches still evolved under purifying selection or neutrality. Again, the most likely model was inferred by LRT and sites detected to be under positive selection at the codon level were manually validated according to alignment quality and reliability.

## Results

A total of 219 members of GT61 gene family were found in *A. thaliana*, *T. cacao*, *V. vinifera*, *C. canephora*, *A. officinalis*, *M. acuminata*, *E. guineensis*, *P. dactylifera*, *A. comosus*, *B. distachyon*, *S. italica*, *O. sativa*, and *A. trichopoda*. In dicots and *A. trichopoda*, the number of GT61 genes found per species ranged between 4 and 7 and in monocots between 9 and 39, with 24 in average (between 15 and 39 when only commelinids are considered, i.e., excluding *A. officinalis*) (Table 1 and Supplementary Data S1).

**Table 1:** Number of sequences per studied species included in main phylogenetic clusters.

Species/group	*G*	*F*	*D*	*A*	Total
*A. thaliana* (ARATH)	–	2	3	2	7
*T. cacao* (THECC)	1	2	1	3	7
*V. vinifera* (VITVI)	1^∗^	1	1	1	4
*C. canephora* (COFCA)	2	–	2	2	6
*A. officinalis* (ASPOF)	1	–	3	5	9
*M. acuminata* (MUSAC)	–	–	2	37	39
*E. guineensis* (ELAGV)	3	–	4	16	23
*P. dactylifera* (PHODA)	1	–	3	11	15
*A. comosus* (ANACO)	1	–	4	23	28
*S. italica* (SETIT)	–	–	5	26	31
*B. distachyon* (BRADI)	–	–	4	17	21
*O. sativa* (ORYSA)	–	–	4	19	23
*A. trichopoda* (AMBTC)	1	2	1	2	6
Total	11	7	37	164	219


*^∗^Probable pseudogene. Species specific five digit code is indicated between brackets.*

### Phylogenetic Analyses

**FIGURE 2:**
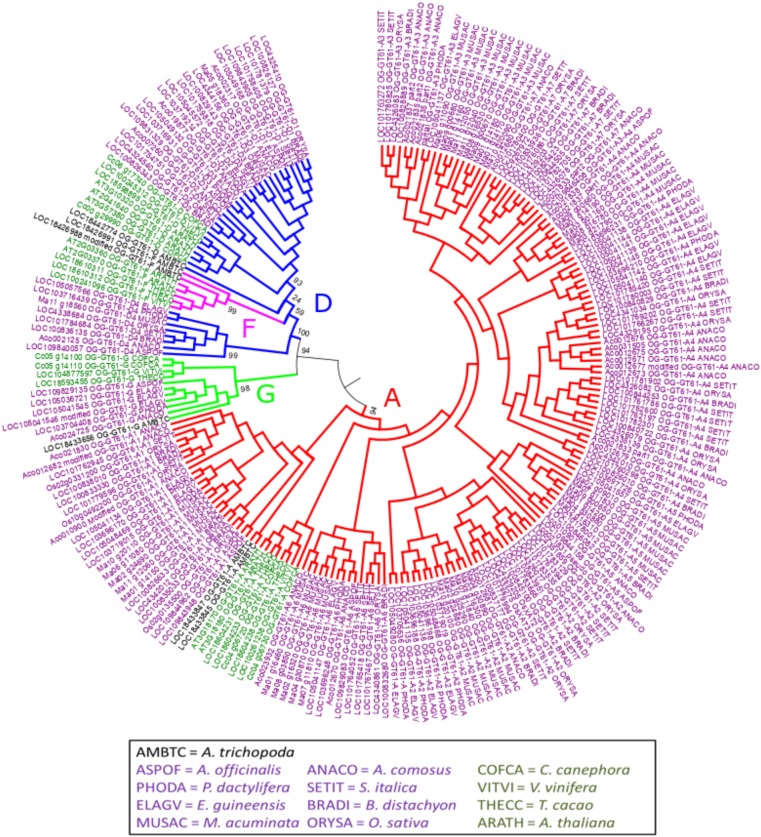
Phylogenetic tree obtained with 219 GT61 sequences [sequences from clade C of Anders et al. (2012) were not analyzed] from 13 species representing angiosperms. Dicot and monocot sequence names are in dark green and purple, respectively. 253 aligned positions were used to build the tree. Branches of sequences assigned to A, D, F, and G orthogroups are indicated in red, blue, fuchsia and green, respectively. Branch aLRT support was indicated only for main branches (complete data are available in Supplementary Data S3).

The phylogenetic tree of the whole GT61 gene family was obtained with 253 aligned positions (amino acids). Three main clades were detected (Figure 2 and Supplementary Data S3). The first one (in green in Figure 2, named clade G), has a strong branch support (aLRT = 0.98) and contains 11 sequences belonging to both monocot and dicot lineages plus one sequence of *A. trichopoda*. However, in this clade, there are no sequences of the following species: *A. thaliana*, *M. acuminata* and all considered Poaceae (Table 1). The second clade is also strongly supported (aLRT = 1), and includes 44 sequences (subclades D and F in Table 1) distributed in sub-clades with variable support (in blue and fuchsia in Figure 2). The third clade (clade A, aLRT = 0.94) is the largest one (in red in Figure 2). It includes 164 sequences, i.e., more than 75% of GT61 sequences (Table 1).

**FIGURE 3:**
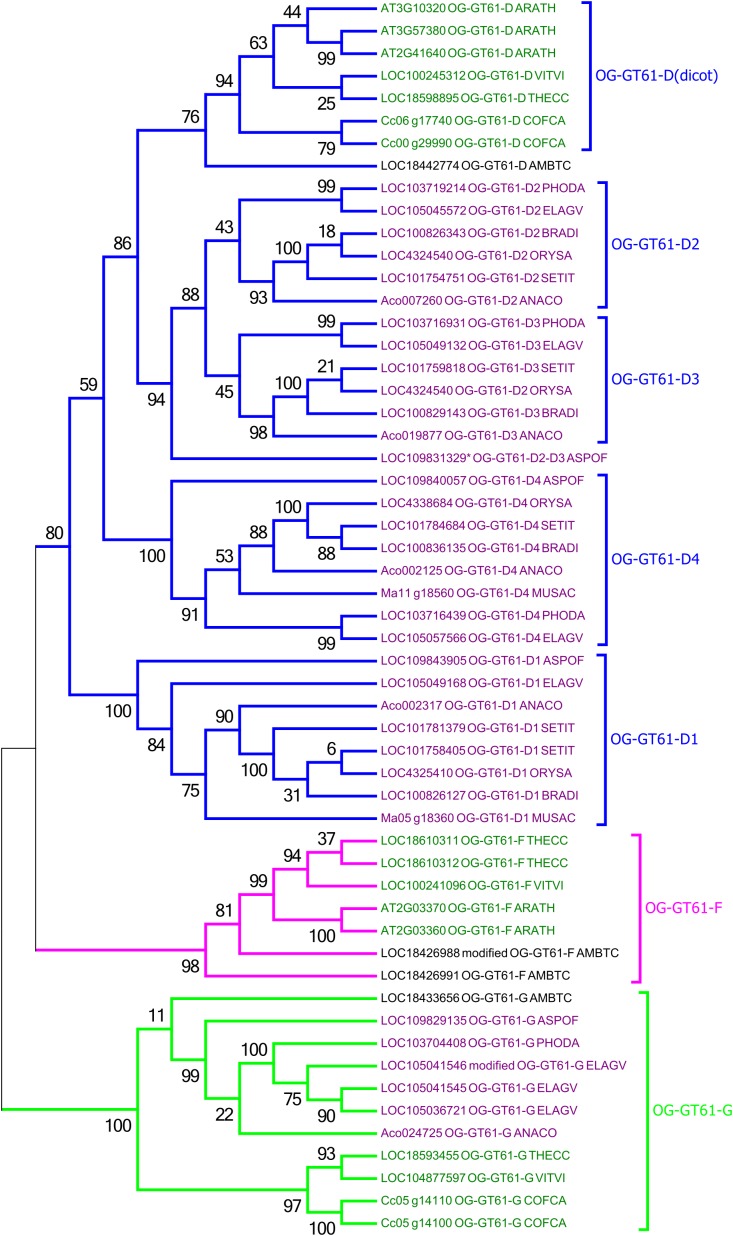
Phylogenetic tree obtained with 55 GT61 sequences from clade D, F, and G. Species origin of sequences was indicated by a five digit code [*A. comosus* (ANACO), *A. officinalis* (ASPOF), *B. distachyon* (BRADI), *E. guineensis* (ELAGV), *M. acuminata* (MUSAC), *O. sativa* (ORYSA), *P. dactylifera* (PHODA), *S. italica* (SETIT), *A. trichopoda* (AMBTC), *A. thaliana* (ARATH), *C. canephora* (COFCA), *T. cacao* (THECC), and *V. vinifera* (VITVI)]. Dicot and monocot sequence names are in dark green and purple, respectively.

A second phylogenetic analysis restricted to the second clade was performed in order to increase its phylogenetic resolution, using the clade G as an outgroup. Thus, the 44 sequences of this clade and 11 of the clade G were re-aligned and the phylogenetic tree was built based on 307 amino acid aligned positions (Figure 3 and Supplementary Data S3). In this new tree, a strongly supported clade (aLRT = 0.98) includes seven sequences from only *A. trichopoda* and three dicots species (*T. cacao*, *V. vinifera*, and *A. thaliana*; in fuchsia in Figures 2, 3). The remaining 34 sequences (aLRT = 0.80) form five new clades named D1 to D4, and D (dicot) (Figure 3). The OG-GT61-D (dicot) clade (aLRT = 0.94) contains all and only dicot sequences (Figure 3). An *A. trichopoda* sequence is branched to this clade with a lower branch support (aLRT = 0.76) (Figure 3). The sequences composing the four remaining clades (D1–D4) come all exclusively from monocot species, and each clade includes sequences from almost all the commelinid species studied. As expected, *A. officinalis* sequences have a basal position in all four clades, with LOC109843905 and LOC109840057 clearly included in clade D1 and D4, respectively, whereas the *A. officinalis* sequence (LOC109831329^∗^) is branched on a basal position of the two clades D2 and D3 (Figure 3).

**FIGURE 4:**
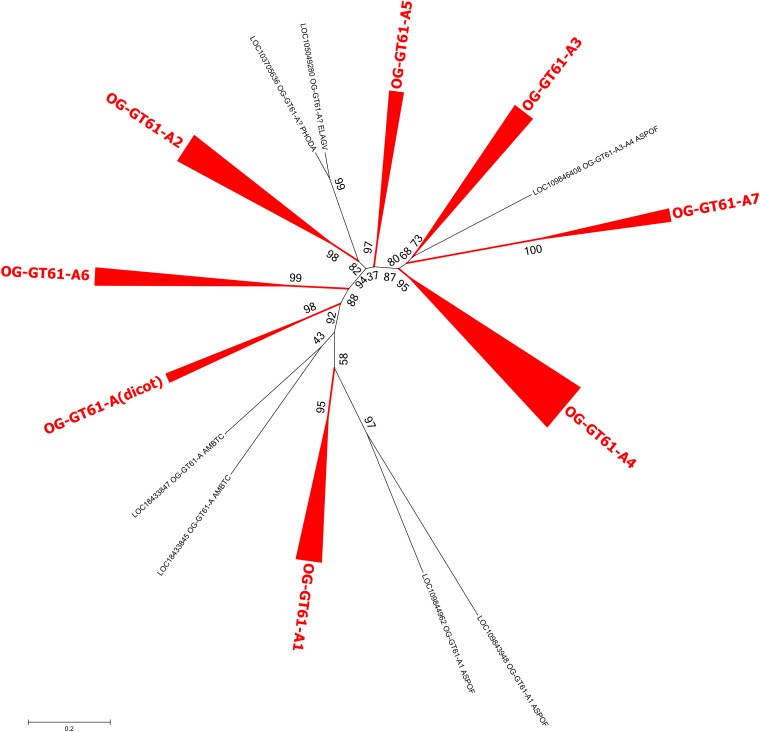
Phylogenetic tree obtained with 164 GT61 sequences from clade A. Clades corresponding to orthogroups are collapsed. The fully expanded representation of the tree is in Supplementary Figure S1.

In the same order of idea, a third phylogenetic tree was built to better disentangle the phylogenetic relationships within the group A. The 164 sequences it contained were re-aligned and a new phylogenetic tree was build based on 277 aligned amino acid positions. Several well-supported clades were identified but relationships among them remained poorly resolved (Figure 4 and Supplementary Data S3). All the dicot sequences were grouped in the same well-supported clade named ‘A (dicot)’. Five others clades contain at least one sequence of each monocot species sampled in our study (A1, A2, A3, A4, and A6) whereas a sixth (A5) is missing sequences from *O. sativa*. Finally, the clade A7 contains only sequences from *A. comosus* and from the three Poaceae species. All these clades have an aLRT support higher than 0.95 except A3 (0.81). The seven remaining sequences, whether grouped or not in small well-supported clades, could not be assigned to the main clades previously described.

### Orthogroups Identification and Classification of GT61 Genes

As previously mentioned, an orthogroup (i.e., group of orthologous genes) is defined, for a given sample of species, as a group of genes all issued from a single ancestral gene present in the last common ancestor of the species being considered. Based on the species considered in this study (i.e., monocots and dicots), we identified 4 orthogroups. The sequences included in the phylogenetic clade G appear clearly derived from the same ancestral gene present in the monocot/dicot last common ancestor and constitutes thus a first orthogroup (named OG-GT61-G). Indeed, the internal topology of clade G follows almost perfectly the expected topology of angiosperm phylogeny. Clade G is well supported and contains sequences from both monocot and dicot species (Figure 3); one sequence from *A. trichopoda* is included in this cluster. Two other orthogroups (OG-GT61-F and OG-GT61-D) were defined in the second main clade. Again, the ancestral gene, from which all the genes of the clade OG-GT61-F are derived, was probably present in the common ancestor of monocot and dicots. However, in this clade, the absence of genes from the monocots species studied here can be interpreted as a gene loss that probably occurred early in the evolution of monocot lineages. Conversely, for the OG-GT61-D, in the lineage of commelinids copy amplification took place that generated four copies; each one was conserved in the descendant species and formed four commelinid-specific orthogroups, named from OG-GT61-D1 to OG-GT61-D4 (Figure 3). The position of the three *A. officinalis* sequences in the phylogenetic tree suggests that the duplication which resulted in the presence of D2 and D3 sequences occurred after commelinids lineage diverged from Asparagales (Figure 3). Due to the low branch support for OG-GT61-D2 and -D3 clades, one cannot exclude the possibility that Acorales and Poales sequence duplications (no representative sequence was found for *M. acuminata*) were independent and successive to the respective lineages separation (Figure 3), implying the existence of a unique orthogroup.

All the sequences of the group A, issued from both dicot and monocot species, were assigned to an orthogroup named OG-GT61-A. Clade A (dicot) contains all the dicots sequences and each dicot species possesses one to three sequences. Species-specific tandem duplications occurred in some species, such as *A. thaliana* and *T. cacao*. A lineage-specific amplification was also observed in *A. trichopoda*. The phylogenetic analysis performed on the sequences of this orthogroup shows that several amplifications occurred but concerned almost exclusively genes from the monocots.

The monocot sequences of OG-GT61-A are more numerous and can be grouped into six clades (A1–A6) containing each all commelinids, and a seventh one (A7), Poales-specific (Figure 4 and Supplementary Figure S1). The positions of *A. officinalis* sequences are, however, sometimes not well-resolved, especially LOC109846408, which is closely related to the GT61-A3, -A4 and -A7 orthogroups. Seven orthogroups commelinid-specific can therefore be defined, and named OG-GT61-A1 to OG-GT61-A7 (Figure 4). Additional amplifications were observed in some commelinid OGs. Internal clades containing each of the three Poaceae species (*O. sativa*, *B. distachyon*, and *S. italica*) were identified: 4 for OG-GT61-A1, 3 for OG-GT61-A2, 5 for OG-GT61-A4 and 3 for OG-GT61-A7. We finally further refined the nomenclature within Poaceae-specific OGs with a letter as a suffix (e.g., OG-GT61-A7a, OG-GT61-A7b, and OG-GT61-A7c, Supplementary Figure S1).

### Genome Distribution of GT61 Sequences

The analysis of the genomic positions of the GT61 sequences from the orthogroup A in monocots showed that several genes are tandemly distributed. Each tandem is composed of genes, each one belonging to a different commelinid-specific OG-A. Moreover, the tandem organization is collinear among the studied monocot species (Table 2). The tandem repeat loci present in the genome of *P. dactylifera* and *E. guineensis* contain members from all the subclades (A1-A6). Differently, in *M. acuminata*, whose genome experienced three lineage-specific WGDs, the number of OG-GT61-A tandem loci is higher (nine loci) and it appears that each of them underwent gene loss, a process known as ‘fractionation process’ in polyploid species (Langham et al., 2004). Thus, the complete ancestral structure of the tandem repeat locus of *M. acuminata* can be inferred by consensus from all the clusters (Table 2). In Poales (not shown in Table 2), because of (i) the presence of the A7 orthogroup members and (ii) the occurrence of additional tandem amplifications, the OG-GT61-A tandem loci harbor a more complex organization, but they still remain partially collinear with those of Arecales and Zingiberales.

**Table 2:** Collinearity in tandem cluster loci of monocot OG-GT61-A genes.

Species/sequence	A1	A2	A3	A4	A5	A6
*P. dactylifera*					
NW_008246516.1	LOC103696170	LOC103696188, LOC103696198	Partial	LOC103696231, LOC103696210	LOC103697726	LOC103696248
NW_008246831.1	LOC103719015	LOC103719019				
*E. guineensis*					
NC_025995.1	LOC105041134	LOC105041135, LOC105041136	LOC105041137	LOC105041138, LOC105041140, LOC105041139, LOC105041141, LOC105041144, LOC105041142, LOC105041145	LOC105041157	LOC105041146
*M. acuminata*					
Chr01	Ma01_g14150		Ma01_g14160, Ma01_g14170, Ma01_g14180	Ma01_g14190	Ma01_g14200	
Chr02		Ma02_g16300			Ma02_g16310	Ma02_g16320
Chr02	Ma02_g24690	Ma02_g24680				
Chr04		Ma04_g30890			Ma04_g30880	Ma04_g30870
Chr06	Ma06_g11080		Ma06_g11090			
Chr07		Ma07_g11840			Ma07_g11830	Ma07_g11810
Chr09			Ma09_g00670, Ma09_g00680	Ma09_g00660	Ma09_g00650	
Chr10	Ma10_g20110	Ma10_g20100	Ma10_g20090			
Chr11	Ma11_g12060	Ma11_g12050	Ma11_g12040			
**Consensus**	**>>>>>>>>>>**	**<<<<<<<<<<**	**<<<<<<<<<<**	**<<<<<<<<<<**	**<<<<<<<<<<**	**>>>>>>>>>>**


*Gene names in green or in red colors indicates forward or reverse relative orientation.*

**FIGURE 5:**
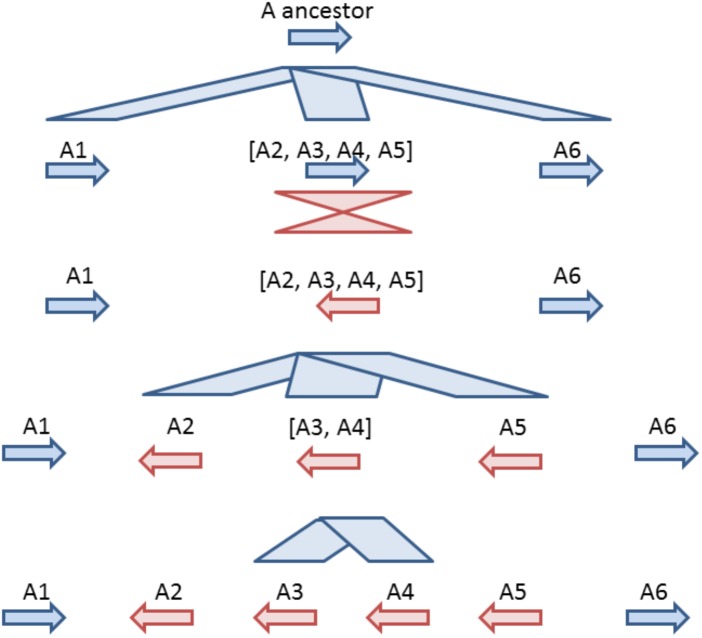
Model for tandem expansion of GT61 genes (symbolized by arrows) belonging to OG-GT61-A in the Commelinid lineage. Blue color indicates tandem duplication, red color indicates inversions. The first step was the tandem triplication of the common GT61-A ancestor followed by inversion of central copy that finally underwent to additional tandem duplication. Succession of second (inversion) and third step (tandem duplication) could be inverted, making the A2, [A3, A4] and A5 amplification predating the inversion of their common ancestor.

Based on comparative analysis of loci on which GT61 genes are tandemly distributed and on the phylogenetic analysis, a model was built to reconstruct the history of GT61 gene amplification before the commelinid radiation. Since the GT61 family underwent additional copy amplifications in the Poales, only *Arecales* and *Zingiberales* are taken into account (Figure 5).

### Selective Pressure in GT61 Family

**FIGURE 6:**
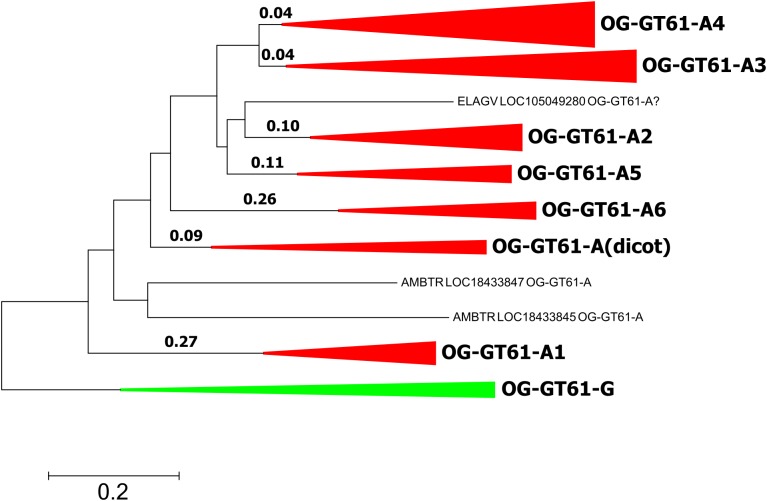
Phylogenetic tree used for PAML analyses. Length is reported for each tested branch. Clades corresponding to orthogroups are collapsed.

In order to investigate evolutionary forces which could explain the diversification of this gene family into six orthogroups in commelinids, several models of codon evolution were tested. Due to the further gene expansion that took place in Poaceae, only *A. comosus* was included in analyses as a species representative of Poales. *A. officinalis* sequences were also removed to simplify the focus on commelinids. The sequences of the OG-GT61-G were included as outgroup. The 103 amino acid sequences were aligned and converted back into codons (311 positions). The phylogenetic tree used in the PAML analysis was built with the same codon alignment (Figure 6 and Supplementary Data S3).

The first model tested the diversification between paralogous sequences within each species and revealed no signal of positive selection (Table 3).

**Table 3:** Analysis for positive selection footprint in paralogous sequences (site models).

		M8a (null) model		M8 model (positive selection)		LRT
Species	# sequences	# parameters	lnL	# parameters	lnL	(M8 vs. M8a)
*A.* *comosus*	23	47	–13824.746	48	–13822.802	0.0486
*E. guineensis*	16	33	–8995.103	34	–8994.781	0.4220
*M. acuminata*	37	75	–21533.671	76	–21533.671	1
*P. dactylifera*	10	21	–7158.605	22	–7158.294	0.4300


The second model allowed us to test the significance of a different dN/dS value on each of the six ancestral branches leading to the different orthogroups (A1–A6), compared to the dN/dS values of the rest of the tree (Figure 6 and Table 4). This analysis showed that on the branch leading to the orthogroup A6, the dN/dS value was significantly higher (0.7543) than the dN/dS values of all the remaining (i.e., background and root) branches of the tree (0.1467 and 0.1881 for the root). This result shows that the constraints were much more relaxed, on average, in this branch, i.e., after the duplication leading to the A6 copy and before the speciation occurred.

**Table 4:** Analysis for positive selection footprint on the A1 to A6 orthologous subgroups branches (‘branch’ models).

Model or tested branch	# parameters	lnL	LRT branch vs. null	ω value (root)	ω value (background)	ω tested branch
Null model (root)	206	–56465.344				
A1	207	–56462.935	0.0282	0.1904	0.1456	0.0974
A2	207	–56463.354	0.0461	0.1890	0.1463	n.e.
A3	207	–56464.683	0.2503	0.1891	0.1463	0.3752
A4	207	–56465.328	0.8577	0.1894	0.1463	0.2104
A5	207	–56463.471	0.0530	0.1888	0.1459	0.6359
A6	207	–56459.395	**5.62** **10^-4^**	0.1881	0.1467	0.7543


*Significant values are in bold.*

To go further in the investigation of the selective constraints on those six branches, other models were used. They tested for the presence of sites under positive selection on the selected branches (Figure 6 and Table 5). On two branches, A5 and A6, a proportion of sites (2.3 and 9.8% for A5 and A6, respectively) were under positive selection. For the A5 branch, the estimated value of dN/dS in the site category expected to be superior to one is exactly 1, suggesting that the significance of the ‘branch-site’ model compared to the null model may be artefactual. On the contrary, the dN/dS value estimated for positively selected sites on the A6 branch was 5.98, confirming that those sites are clearly under positive selection on this branch. Indeed, Bayes empirical method (BEB) showed that 5 codons have a high posterior probability to be under positive selection (*p* > 0.95). These codons are located in the cleaned alignment positions 67, 129, 151, 236, and 311, where amino acid variants are often specific to the OG-GT61 sequences (Supplementary Data S4).

**Table 5:** Analysis for positive selection footprint on the A1 to A6 orthologous subgroups branches (‘branch-site’ models).

	Null model				Branch-site model			
Tested branch	# parameters	lnL	*p*_0_ ω_0_ < 1	*p*_1_ (ω_1_ = 1)	# parameters	lnL	Tested branch *p*_2_ ω_2_> 1	LRT branch-site vs. null
A1	207	–55746.823	0.81 ω_0_ = 0.166	0.19	208	–55746.823	n.e.	1
A2	207	–55727.382	0.81 ω_0_ = 0.167	0.19	208	–55727.382	n.e.	n.e.
A3	207	–55728.298	0.81 ω_0_ = 0.167	0.19	208	–55728.946	n.e.	n.e.
A4	207	–55728.946	0.81 ω_0_ = 0.167	0.19	208	–55728.946	n.e.	0,9980
A5	207	–55733.021	0.81 ω_0_ = 0.167	0.19	208	–55728.925	0.023 ω_2_ = 1	**0.421** **10^-2^**
A6	207	–55721.491	0.81 ω_0_ = 0.166	0.19	208	–55717.905	0.098 ω_2_ = 5.98	**0.741 10**^-^**^2^**


*Significant values are in bold.*

## Discussion

In order to reconstruct the evolutionary history of the GT61 gene family in angiosperms and to propose a classification of its members, we conducted a deep phylogenetic analysis of the GT61 genes present in 13 species representative of angiosperms. Our strategy was to identify orthogroups (OG) for three different sub-sample of species, from the widest one (monocots and dicots) to the narrowest (Poaceae species), with an intermediate one (commelinid species). This strategy allowed us to make assumptions on how many ancestral genes were present at each node of interest across the angiosperm evolutionary history, and thus to model when and where duplication and loss events occurred.

When all the species were considered (the largest species sub-sample), four orthogroups could be defined: OG-GT61-A, -D, -F, and -G [the very divergent clade C in the phylogeny of Anders et al. (2012) was not considered in this study]. It indicates that all the GT61 genes from monocots and dicots derived from at least 4 ancestral genes. This number is consistent with the fact that six GT61 genes were found in *A. trichopoda*, included here as an outgroup for the monocot/dicot lineage. Actually, two of them are included in the clade containing the OG-GT61-A genes and probably result from an *A. trichopoda* lineage-specific duplication while other two are included in the well-supported F orthogroup (Figures 2–4).

Since a commelinid-specific expansion was observed within OG-GT61-A and -D, the commelinids were chosen as the second species sub-sample to define narrower OGs. Thus, 7 and 4 orthogroups, respectively (OG-GT61-A1 to -A7 and OG-GT61-D1 to D4) were identified. Finally, due to additional amplifications detected in A orthogroups of Poaceae (represented by *O. sativa*, *B. distachyon* and *S. italica* in our study), Poaceae constituted the third species level and 15 additional OG could be defined (Supplementary Figure S1). This number should be validated by an analysis including a more complete representation of Poaceae species. A GT61 additional amplification was observed also in Arecales, but since only two species represented this group, we considered unreliable to define an Arecales-specific orthogroups.

The GT61 gene family underwent dramatic copy amplifications, as already noted in the study by Anders et al. (2012), which was restricted to Poaceae species, in particular within OG-GT61-A. However, the estimation of when the amplifications occurred, in particular over the course of monocot evolution needed to be specified. Our results show that a first round of amplification involved the ancestor of all commelinids analyzed here. Moreover, the phylogenetic positions of the non-commelinid *A. officinalis* GT61 sequences in D and A orthogroups suggests that the amplification process started before the split between the Asparagales and Commelinid lineages. The copy number amplification observed for grasses (Poaceae) by Anders et al. (2012) would actually involve a larger taxonomic range (at least for some duplication events).

Gene amplifications shared by the commelinid were observed in two different orthogroups: OG-GT61-A and, to a lesser extent, OG-GT61-D. The dicot clade position in the OG-GT61-A and OG-GT61-D trees raises the question whether the amplification process started before the monocot/dicot split and was followed by loss of the duplicated copies in the dicot lineage. For example GT61-A1 clade appears to have a basal position in the OG-GT61-A subtree (Figure 2 and Supplementary Figure S1) but unresolved position of the outgroup (*A. trichopoda*) sequences prevent us from solving this question.

The Poales-specific amplifications within the orthogroups A1, A2, A4, and A7 indicate that new copies were retained. With the exception of the Poales-specific A7 OG, independent amplifications took place also in Arecales (*P. dactylifera* and *E. guineensis*). Finally, in the *M. acuminata* GT61 family, gene amplification was observed in all A OGs, likely explained by the three WGDs that took place during the *Musa* genome evolution (D’Hont et al., 2012). Taken together, these observations indicate that the expansion in the GT61 family was not an evolutionary burst that occurred before the Commelinids or Poaceae radiation but rather the result of recurrent duplication events in the monocot evolution. The analysis of GT61 gene genomic locations indicates that the GT61 family expansion was shaped by both local tandem amplification and large scale duplications (WGDs).

Combined analyses of phylogeny and comparative genomics (Table 2) of the OG-GT61-A members allowed us to reconstruct the first phase of amplification, i.e., the one concerning the entire commelinid lineage (Figure 5). This reconstruction is based on highly supported phylogenetic branches (aLRT higher than 0.9) and on relative positions and orientations of GT61 genes in tandemly organized loci found in all the commelinids. However, to reach a higher level of resolution in the GT61 family tandem amplification history in monocots, additional high quality genome would be needed, especially in other monocot orders such as Acorales or Liliales for instance.

One hypothesis to explain the retention of duplicated copies during the genome evolution of all monocot species analyzed here is that the GT61 genes underwent a functional divergence. This hypothesis relies, in a schematic way, either on the neofunctionalization scenario under which a new advantageous function appears on one copy (for which positive selection is expected to act), or on the subfunctionalization scenario under which divergence accumulated between copies makes them non-redundant and consequently prevents each of them from being eliminated during the evolution (for which only neutral processes are expected) (Moore and Purugganan, 2005; Innan and Kondrashov, 2010). In order to go further, and to figure out which functional implications could be deduced from the evolutionary history of GT61 gene family, we searched for putative footprints of selection. The PAML analysis performed on the sequences of OG-GT61-A of monocots revealed positive selection footprints on the branch specific to the OG-GT61-A6. In particular, five codons with a dN/dS value significantly higher than 1 were identified. Only two of them lie in the DUF563 domain. No other branches or sites under positive selection were identified but action of diversifying selection cannot be excluded. In particular, some analyzed branches are really short (for example in the case of OG-GT61-A3 and -A4, Figure 6) and the statistical power is known to be reduced in short branches. It is also possible that the alignment cleaning steps removed significant codons.

At present, the function of only three OG-GT61-A genes has been identified: TaXAT1 and TaXAT2 in wheat (Anders et al., 2012) and XAX1 in rice (Chiniquy et al., 2012). These three genes are involved in cell wall xylan synthesis. TaXAT1 and TaXAT2 belong to the orthogroups GT61-A1a and GT61-A2b, respectively; the rice XAX1 belongs to the commelinid orthogroup GT61-A7 (Poales specific). It is likely that other copies share similar function in synthesis of cell wall xylans. The increasing number of genes observed in the monocot could be related to the higher biochemical complexity and diversity of the cell wall of this taxonomic group compared to other plant species (Peña et al., 2016). Duplicated copies could have enabled the transfer of different molecules to the xylose chain, thus modifying the cell wall composition. Moreover, the cell wall xylan composition is different in tissues and organs (Anders et al., 2012) and a part of the amplification could be followed by regulatory subfunctionalization adjusting expression of the cell wall network genes to an optimal level in each plant tissue. Since these additional copies were not lost during the course of evolution, this retention possibly being the result of selective forces, the new functions most probably improved the fitness of the plants.

## Author Contributions

AC and MR conceived the study. AC performed the analyses and wrote the manuscript. NC performed the PAML analysis.

## Conflict of Interest Statement

The authors declare that the research was conducted in the absence of any commercial or financial relationships that could be construed as a potential conflict of interest.

## Supplementary Material

Figure S1 Fully expanded representation of unrooted phylogenetic tree of clade A. Dicot and monocot sequence names are in dark green and purple, respectively. Orthogroups based on commelinid and Poaceae ancestors are reported in red and in brown, respectively. Species origin of sequences was indicated by a five digit code [*A. comosus* (ANACO), *A. officinalis* (ASPOF), *B. distachyon* (BRADI), *E. guineensis* (ELAGV), *M. acuminata* (MUSAC), *O. sativa* (ORYSA), *P. dactylifera* (PHODA), *S. italica* (SETIT), *A. trichopoda* (AMBTC), *A. thaliana* (ARATH), *C. canephora* (COFCA), *T. cacao* (THECC), and *V. vinifera* (VITVI)].

DATA S1 Lists of GT61 genes in the 13 analyzed species (*A. comosus*, *A. officinalis*, *B. distachyon*, *E. guineensis*, *M. acuminata*, *O. sativa*, *P. dactylifera*, *S. italica*, *A. trichopoda*, *A. thaliana*, *C. canephora*, *T. cacao*, and *V. vinifera*).

DATA S2 Sequences of 219 GT61 genes used in this study.

DATA S3 Newick format of phylogenetic trees obtained with all GT61 sequences (219), with sequences in clades D, G and F (55) and clade A (164).

DATA S4 Alignment of GT61-A and -G sequence regions for PAML analyses.

## References

[B1] Anders N. Wilkinson M. D. Lovegrove A. Freeman J. Tryfona T. Pellny T. K. (2012). Glycosyl transferases in family 61 mediate arabinofuranosyl transfer onto xylan in grasses. *Proc. Natl. Acad. Sci. U.S.A.* 109 989–993. 10.1073/pnas.1115858109 22215597 PMC3271882

[B2] Berardini T. Z. Reiser L. Li D. Mezheritsky Y. Muller R. Strait E. (2015). The Arabidopsis information resource: making and mining the “gold standard” annotated reference plant genome. *Genesis* 53 474–485. 10.1002/dvg.22877 26201819 PMC4545719

[B3] Castresana J. (2000). Selection of conserved blocks from multiple alignments for their use in phylogenetic analysis. *Mol. Biol. Evol.* 17 540–552. 10.1093/oxfordjournals.molbev.a026334 10742046

[B4] Cenci A. Guignon V. Roux N. Rouard M. (2014). Genomic analysis of NAC transcription factors in banana (*Musa acuminata*) and definition of NAC orthologous groups for monocots and dicots. *Plant Mol. Biol.* 85 63–80. 10.1007/s11103-013-0169-2 24570169 PMC4151281

[B5] Cenci A. Rouard M. (2017)). Evolutionary analyses of GRAS transcription factors in angiosperms. *Front. Plant Sci.* 8:273. 10.3389/fpls.2017.00273 28303145 PMC5332381

[B6] Chiniquy D. Sharma V. Schultink A. Baidoo E. E. Rautengarten C. Cheng K. (2012). XAX1 from glycosyltransferase family 61 mediates xylosyltransfer to rice xylan. *Proc. Natl. Acad. Sci.U.S.A.* 109 17117–17122. 10.1073/pnas.1202079109 23027943 PMC3479505

[B7] Coutinho P. M. Deleury E. Davies G. J. Henrissat B. (2003). An evolving hierarchical family classification for glycosyltransferases. *J. Mol. Biol.* 328 307–317. 10.1016/S0022-2836(03)00307-3 12691742

[B8] De Mita S. Siol M. (2012). EggLib: processing, analysis and simulation tools for population genetics and genomics. *BMC Genet.* 13:27. 10.1186/1471-2156-13-27 22494792 PMC3350404

[B9] Dereeper A. Bocs S. Rouard M. Guignon V. Ravel S. Tranchant-Dubreuil C. (2015). The coffee genome hub: a resource for coffee genomes. *Nucleic Acid Res.* 43 D1028–D1035. 10.1093/nar/gku1108 25392413 PMC4383925

[B10] Dereeper A. Guignon V. Blanc G. Audic S. Buffet S. Chevenet F. (2008). Phylogeny.fr: robust phylogenetic analysis for the non-specialist. *Nucleic Acids Res.* 36 W465–W469. 10.1093/nar/gkn180 18424797 PMC2447785

[B11] D’Hont A. Denoeud F. Aury J.-M. Baurens F.-C. Carreel F. Garsmeur O. (2012). The banana (Musa acuminata) genome and the evolution of monocotyledonous plants. *Nature* 488 213–217. 10.1038/nature11241 22801500

[B12] Droc G. Lariviere D. Guignon V. Yahiaoui N. This D. Garsmeur O. (2013). The Banana genome hub. *Database* 2013:bat035. 10.1093/database/bat035 23707967 PMC3662865

[B13] Fischer I. Dainat J. Ranwez V. Glémin S. Dufayard J.-F. Chantret N. (2014). Impact of recurrent gene duplication on adaptation of plant genomes. *BMC Plant Biol.* 14:151. 10.1186/1471-2229-14-151 24884640 PMC4049390

[B14] Fischer I. Diévart A. Droc G. Dufayard J.-F. Chantret N. (2016). Evolutionary Dynamics of the leucine-rich repeat receptor-like kinase (LRR-RLK) subfamily in Angiosperms. *Plant Physiol.* 170 1595–1610. 10.1104/pp.15.01470 26773008 PMC4775120

[B15] Freeman J. Ward J. L. Kosik O. Lovegrove A. Wilkinson M. D. Shewry P. R. (2017). Feruloylation and structure of arabinoxylan in wheat endosperm cell walls from RNAi lines with suppression of genes responsible for backbone synthesis and decoration. *Plant Biotechnol. J.* 15 1429–1438. 10.1111/pbi.12727 28316134 PMC5633762

[B16] Guindon S. Delsuc F. Dufayard J.-F. Gascuel O. (2009). Estimating maximum likelihood phylogenies with PhyML. *Methods Mol. Biol.* 537 113–137. 10.1007/978-1-59745-251-9_6 19378142

[B17] Guindon S. Gascuel O. (2003). A simple, fast, and accurate algorithm to estimate large phylogenies by maximum likelihood. *Syst. Biol.* 52 696–704. 10.1080/10635150390235520 14530136

[B18] Innan H. Kondrashov F. (2010). The evolution of gene duplications: classifying and distinguishing between models. *Nat. Rev. Genet.* 11 97–108. 10.1038/nrg2689 20051986

[B19] Katoh K. Standley D. M. (2013). MAFFT multiple sequence alignment software version 7: improvements in performance and usability. *Mol. Biol. Evol.* 30 772–780. 10.1093/molbev/mst010 23329690 PMC3603318

[B20] Langham R. J. Walsh J. Dunn M. Ko C. Goff S. A. Freeling M. (2004). Genomic duplication, fractionation and the origin of regulatory novelty. *Genetics* 166 935–945. 10.1534/genetics.166.2.935 15020478 PMC1470742

[B21] Li W. Cowley A. Uludag M. Gur T. McWilliam H. Squizzato S. (2015). The EMBL-EBI bioinformatics web and programmatic tools framework. *Nucleic Acids Res.* 43 W580–W584. 10.1093/nar/gkv279 25845596 PMC4489272

[B22] Lombard V. Golaconda Ramulu H. Drula E. Coutinho P. M. Henrissat B. (2014). The carbohydrate-active enzymes database (CAZy) in 2013. *Nucleic Acids Res.* 42 D490–D495. 10.1093/nar/gkt1178 24270786 PMC3965031

[B23] Mitchell R. A. C. Dupree P. Shewry P. R. (2007). A novel bioinformatics approach identifies candidate genes for the synthesis and feruloylation of arabinoxylan. *Plant Physiol.* 144 43–53. 10.1104/pp.106.094995 17351055 PMC1913792

[B24] Moore R. C. Purugganan M. D. (2005). The evolutionary dynamics of plant duplicate genes. *Curr. Opin. Plant Biol.* 8 122–128. 10.1016/j.pbi.2004.12.001 15752990

[B25] Peña M. J. Kulkarni A. R. Backe J. Boyd M. O’Neill M. A. York W. S. (2016). Structural diversity of xylans in the cell walls of monocots. *Planta* 244 589–606. 10.1007/s00425-016-2527-1 27105886

[B26] Phan J. L. Tucker M. R. Khor S. F. Shirley N. Lahnstein J. Beahan C. (2016). Differences in glycosyltransferase family 61 accompany variation in seed coat mucilage composition in Plantago spp. *J. Exp. Bot.* 67 6481–6495. 10.1093/jxb/erw424 27856710 PMC5181589

[B27] Rini J. Esko J. Varki A. (2009). “Glycosyltransferases and glycan-processing Enzymes,” in *Essentials of Glycobiology*, eds Varki A. Cummings R. D. Esko J. D. Freeze H. H. Stanley P. Bertozzi C. R. (Cold Spring Harbor NY: Cold Spring Harbor Laboratory Press). 20301247

[B28] Rouard M. Guignon M. Aluome C. Laporte M.-A. Droc G. Walde C. (2010). GreenPhylDB v2.0: comparative and functional genomics in plants. *Nucleic Acids Res.* 39 D1095–D1102. 10.1093/nar/gkq811 20864446 PMC3013755

[B29] Strasser R. Mucha J. Mach L. Altmann F. Wilson I. B. H. Glössl J. (2000). Molecular cloning and functional expression of β1,2-xylosyltransferase cDNA from *Arabidopsis thaliana*. *FEBS Lett.* 472 105–108. 10.1016/S0014-5793(00)01443-5 10781814

[B30] Tamura K. Stecher G. Peterson D. Filipski A. Kumar S. (2013). MEGA6: molecular evolutionary genetics analysis version 6.0. *Mol. Biol. Evol.* 30 2725–2729. 10.1093/molbev/mst197 24132122 PMC3840312

[B31] Van Bel M. Diels T. Vancaester E. Kreft L. Botzki A. Van de Peer Y. (2018). PLAZA 4.0: an integrative resource for functional, evolutionary and comparative plant genomics. *Nucleic Acids Res.* 46 D1190–D1196. 10.1093/nar/gkx1002 29069403 PMC5753339

[B32] Vogel J. (2008). Unique aspects of the grass cell wall. *Curr. Opin. Plant Biol.* 11 301–307. 10.1016/j.pbi.2008.03.002 18434239

[B33] Voiniciuc C. Günl M. Schmidt M. H.-W. Usadel B. (2015). Highly branched xylan made by irregular xylem14 and mucilage-related21 links mucilage to arabidopsis Seeds. *Plant Physiol.* 169 2481–2495. 10.1104/pp.15.01441 26482889 PMC4677919

[B34] Yang Z. (1997). PAML: a program package for phylogenetic analysis by maximum likelihood. *Bioinformatics* 13 555–556. 10.1093/bioinformatics/13.5.555 9367129

