# Supplementary figures and images for: Glycosyltransferase Family 61 in Liliopsida (Monocot): The Story of a Gene Family Expansion
Source: Front Plant Sci. 2018 Dec 11;9:1843. doi: 10.3389/fpls.2018.01843 (PMC6297846; doi:10.3389/fpls.2018.01843)

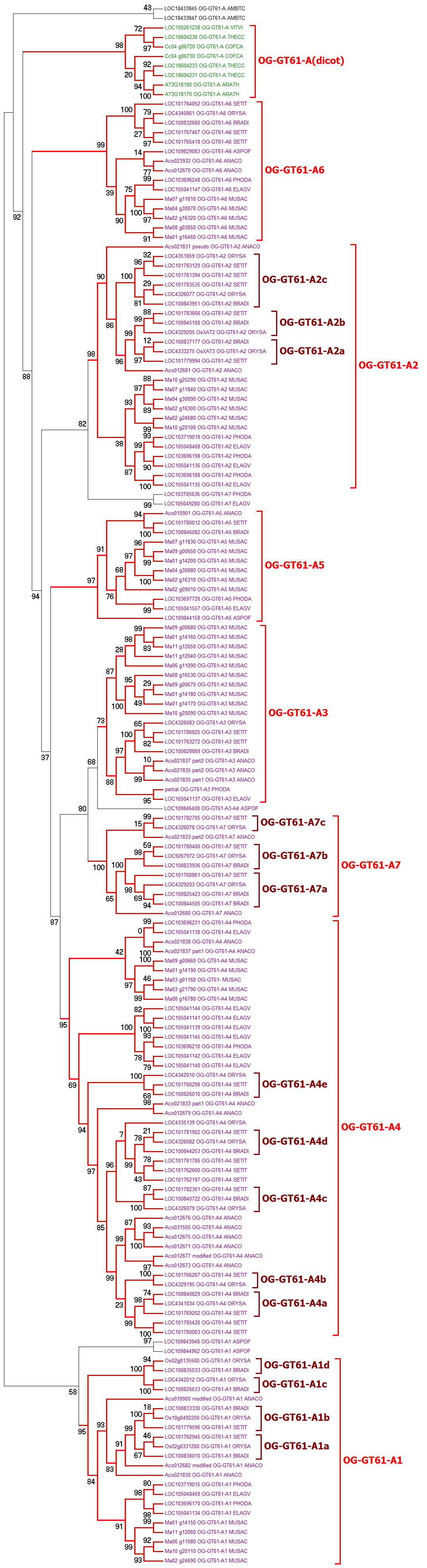

Supplement: Figure S1 — Fully expanded representation of unrooted phylogenetic tree of clade A. Dicot and monocot sequence names are in dark green and purple, respectively. Orthogroups based on commelinid and Poaceae ancestors are reported in red and in brown, respectively. Species origin of sequences was indicated by a five digit code [A. comosus (ANACO), A. officinalis (ASPOF), B. distachyon (BRADI), E. guineensis (ELAGV), M. acuminata (MUSAC), O. sativa (ORYSA), P. dactylifera (PHODA), S. italica (SETIT), A. trichopoda (AMBTC), A. thaliana (ARATH), C. canephora (COFCA), T. cacao (THECC), and V. vinifera (VITVI)]. [file Image_1.TIF]
