# Supplementary material for: Glycosyltransferase Family 61 in Liliopsida (Monocot): The Story of a Gene Family Expansion
Source: Front Plant Sci. 2018 Dec 11;9:1843. doi: 10.3389/fpls.2018.01843 (PMC6297846; doi:10.3389/fpls.2018.01843)
Supplement: DATA S3 — Newick format of phylogenetic trees obtained with all GT61 sequences (219), with sequences in clades D, G and F (55) and clade A (164). [file Data_Sheet_3.docx]

All 219 sequences (253 aligned positions).

(((((((((((((LOC101763272_OG-GT61-A3_SETIT,(LOC101780825_OG-GT61-A3_SETIT,LOC4326083_OG-GT61-A3_ORYSA)0.2330)0.6640,LOC100828889_OG-GT61-A3_BRADI)1.0000,((Aco021837_part2_OG-GT61-A3_ANACO,Aco021835_part2_OG-GT61-A3_ANACO)0.8110,Aco021835_part1_OG-GT61-A3_ANACO)0.9960)0.9620,(partial_OG-GT61-A3_PHODA,LOC105041137_OG-GT61-A3_ELAGV)0.8370)0.9180,(Ma06_g11090_OG-GT61-A3_MUSAC,(((Ma09_g00680_OG-GT61-A3_MUSAC,(Ma01_g14160_OG-GT61-A3_MUSAC,Ma11_g12050_OG-GT61-A3_MUSAC)0.8650)0.9960,Ma11_g12040_OG-GT61-A3_MUSAC)0.9860,((Ma08_g16530_OG-GT61-A3_MUSAC,((Ma09_g00670_OG-GT61-A3_MUSAC,Ma01_g14180_OG-GT61-A3_MUSAC)0.6620,Ma01_g14170_OG-GT61-A3_MUSAC)0.4360)0.9660,Ma10_g20090_OG-GT61-A3_MUSAC)0.9990)0.4770)0.9040)0.6780,((Aco012680_OG-GT61-A7_ANACO,((((LOC101780400_OG-GT61-A7_SETIT,LOC9267972_OG-GT61-A7_ORYSA)0.6090,LOC100833936_OG-GT61-A7_BRADI)0.9850,(LOC101760861_OG-GT61-A7_SETIT,(LOC4329203_OG-GT61-A7_ORYSA,(LOC100825423_OG-GT61-A7_BRADI,LOC100844505_OG-GT61-A7_BRADI)0.9700)0.4440)0.9730)0.9990,((LOC101782705_OG-GT61-A7_SETIT,LOC4326078_OG-GT61-A7_ORYSA)0.9910,Aco021833_part2_OG-GT61-A7_ANACO)0.8410)0.1840)1.0000,LOC109846408_OG-GT61-A3-A4_ASPOF)0.1150)0.9140,((((Aco021838_OG-GT61-A4_ANACO,Aco021837_part1_OG-GT61-A4_ANACO)1.0000,((Ma09_g00660_OG-GT61-A4_MUSAC,Ma01_g14190_OG-GT61-A4_MUSAC)1.0000,((Ma03_g01160_OG-GT61-A4_MUSAC,Ma08_g16780_OG-GT61-A4_MUSAC)0.3290,Ma03_g21790_OG-GT61-A4_MUSAC)0.9660)0.9580)0.0550,(LOC103696231_OG-GT61-A4_PHODA,LOC105041138_OG-GT61-A4_ELAGV)1.0000)0.1990,(((LOC105041144_OG-GT61-A4_ELAGV,(LOC105041141_OG-GT61-A4_ELAGV,LOC105041139_OG-GT61-A4_ELAGV)1.0000)0.8470,((LOC105041145_OG-GT61-A4_ELAGV,LOC103696210_OG-GT61-A4_PHODA)0.8680,(LOC105041142_OG-GT61-A4_ELAGV,LOC105041140_OG-GT61-A4_ELAGV)0.9510)0.7640)1.0000,(((((((LOC101785420_OG-GT61-A4_SETIT,LOC101780003_OG-GT61-A4_SETIT)1.0000,((LOC100840829_OG-GT61-A4_BRADI,LOC4341034_OG-GT61-A4_ORYSA)0.8450,LOC101769202_OG-GT61-A4_SETIT)0.9880)0.9780,(LOC101766267_OG-GT61-A4_SETIT,LOC4329195_OG-GT61-A4_ORYSA)1.0000)0.7050,((Aco012676_OG-GT61-A4_ANACO,((Aco031505_OG-GT61-A4_ANACO,Aco012675_OG-GT61-A4_ANACO)0.8790,Aco012671_OG-GT61-A4_ANACO)1.0000)0.8870,(Aco012677_modified_OG-GT61-A4_ANACO,Aco012673_OG-GT61-A4_ANACO)0.9180)0.9940)0.9990,(((((LOC101781902_OG-GT61-A4_SETIT,LOC4326082_OG-GT61-A4_ORYSA)0.0490,LOC100844253_OG-GT61-A4_BRADI)0.6520,((LOC101761786_OG-GT61-A4_SETIT,LOC101762600_OG-GT61-A4_SETIT)0.7450,LOC101762197_OG-GT61-A4_SETIT)0.4270)0.9980,((LOC101782301_OG-GT61-A4_SETIT,LOC100840722_OG-GT61-A4_BRADI)0.9530,LOC4326079_OG-GT61-A4_ORYSA)1.0000)0.0800,LOC4335139_OG-GT61-A4_ORYSA)0.0610)0.2680,(Aco021833_part1_OG-GT61-A4_ANACO,Aco012679_OG-GT61-A4_ANACO)0.9890)0.9800,(LOC4342010_OG-GT61-A4_ORYSA,(LOC101760298_OG-GT61-A4_SETIT,LOC100826010_OG-GT61-A4_BRADI)0.7370)1.0000)0.9260)0.7530)0.9610)0.8040,((((LOC103697726_OG-GT61-A5_PHODA,LOC105041557_OG-GT61-A5_ELAGV)0.9950,(Ma02_g09010_OG-GT61-A5_MUSAC,((Ma07_g11830_OG-GT61-A5_MUSAC,(Ma09_g00650_OG-GT61-A5_MUSAC,Ma01_g14200_OG-GT61-A5_MUSAC)1.0000)0.9580,(Ma04_g30880_OG-GT61-A5_MUSAC,Ma02_g16310_OG-GT61-A5_MUSAC)1.0000)0.9630)0.4570)0.7870,((LOC101780012_OG-GT61-A5_SETIT,LOC100846082_OG-GT61-A5_BRADI)1.0000,Aco010901_OG-GT61-A5_ANACO)0.9130)0.9120,LOC109844158_OG-GT61-A5_ASPOF)0.9700)0.6810,(((Aco021831_pseudo_OG-GT61-A2_ANACO,((((LOC4351859_OG-GT61-A2_ORYSA,LOC101783129_OG-GT61-A2_SETIT)0.4170,LOC101761394_OG-GT61-A2_SETIT)0.9860,((LOC101783535_OG-GT61-A2_SETIT,LOC4326077_OG-GT61-A2_ORYSA)0.3730,LOC100843951_OG-GT61-A2_BRADI)0.9060)1.0000,((((LOC101783666_OG-GT61-A2_SETIT,LOC100845100_OG-GT61-A2_BRADI)0.8360,LOC4329205_OsXAT2_OG-GT61-A2_ORYSA)0.9950,(LOC4333275_OsXAT3_OG-GT61-A2_ORYSA,(LOC101779994_OG-GT61-A2_SETIT,LOC100837177_OG-GT61-A2_BRADI)0.4440)0.9480)0.9920,Aco012681_OG-GT61-A2_ANACO)0.9420)0.8770)0.8420,((((Ma04_g30890_OG-GT61-A2_MUSAC,Ma02_g16300_OG-GT61-A2_MUSAC)0.8820,(Ma07_g11840_OG-GT61-A2_MUSAC,Ma10_g25290_OG-GT61-A2_MUSAC)0.9140)0.9460,(Ma02_g24680_OG-GT61-A2_MUSAC,Ma10_g20100_OG-GT61-A2_MUSAC)0.9980)0.8310,(((LOC103719019_OG-GT61-A2_PHODA,LOC105048468_OG-GT61-A2_ELAGV)0.9360,(LOC103696198_OG-GT61-A2_PHODA,LOC105041136_OG-GT61-A2_ELAGV)0.8880)0.9960,(LOC103696188_OG-GT61-A2_PHODA,LOC105041135_OG-GT61-A2_ELAGV)1.0000)0.8480)0.9350)0.9600,(LOC103705636_OG-GT61-A_PHODA,LOC105049280_OG-GT61-A_ELAGV)1.0000)0.8810)0.9020,(((LOC100832680_OG-GT61-A6_BRADI,(LOC4340861_OG-GT61-A6_ORYSA,(LOC101767467_OG-GT61-A6_SETIT,LOC101765418_OG-GT61-A6_SETIT)0.9720)0.3570)0.8930,LOC101764052_OG-GT61-A6_SETIT)1.0000,((((LOC109829083_OG-GT61-A6_ASPOF,Aco012670_OG-GT61-A6_ANACO)0.7380,(LOC103696248_OG-GT61-A6_PHODA,LOC105041147_OG-GT61-A6_ELAGV)0.9970)0.6660,(Ma07_g11810_OG-GT61-A6_MUSAC,(Ma04_g30870_OG-GT61-A6_MUSAC,Ma02_g16320_OG-GT61-A6_MUSAC)0.9850)1.0000)0.9420,((Ma08_g03850_OG-GT61-A6_MUSAC,Ma01_g16460_OG-GT61-A6_MUSAC)0.8470,Aco023932_OG-GT61-A6_ANACO)0.8610)0.9010)1.0000)0.7680,(Cc04_g06730_OG-GT61-A_COFCA,((LOC100261238_OG-GT61-A_VITVI,(LOC18604238_OG-GT61-A_THECC,Cc04_g06720_OG-GT61-A_COFCA)0.9290)0.7520,((LOC18604233_OG-GT61-A_THECC,LOC18604231_OG-GT61-A_THECC)0.8390,(AT3G18180_OG-GT61-A_ARATH,AT3G18170_OG-GT61-A_ARATH)1.0000)0.9630)0.6400)0.9460)0.8890,((LOC18433845_OG-GT61-A_AMBTC,LOC18433847_OG-GT61-A_AMBTC)0.4660,((LOC109843948_OG-GT61-A1_ASPOF,LOC109844962_OG-GT61-A1_ASPOF)0.9370,(((Os02g0135500_OG-GT61-A1_ORYSA,LOC100835033_OG-GT61-A1_BRADI)0.7570,(LOC4342012_OG-GT61-A1_ORYSA,LOC100826633_OG-GT61-A1_BRADI)1.0000)0.9990,((((Ma01_g14150_OG-GT61-A1_MUSAC,Ma11_g12060_OG-GT61-A1_MUSAC)0.9960,(Ma02_g24690_OG-GT61-A1_MUSAC,(Ma06_g11080_OG-GT61-A1_MUSAC,Ma10_g20110_OG-GT61-A1_MUSAC)0.9080)0.9480)0.9720,((LOC103719015_OG-GT61-A1_PHODA,LOC105048469_OG-GT61-A1_ELAGV)0.7570,(LOC103696170_OG-GT61-A1_PHODA,LOC105041134_OG-GT61-A1_ELAGV)0.9830)0.9950)0.9480,(Aco010900_Modified_OG-GT61-A1_ANACO,((((Os10g0492200_OG-GT61-A1_ORYSA,(LOC101779596_OG-GT61-A1_SETIT,LOC100833330_OG-GT61-A1_BRADI)0.0000)1.0000,((LOC100838010_OG-GT61-A1_BRADI,Os02g0331200_OG-GT61-A1_ORYSA)0.5070,LOC101762945_OG-GT61-A1_SETIT)0.8610)0.9920,Aco012682_modified_OG-GT61-A1_ANACO)0.9450,Aco021830_OG-GT61-A1_ANACO)0.6940)0.9330)0.9470)0.9830)0.7440)0.5960)0.9420,(((LOC18433656_OG-GT61-G_AMBTC,(Aco024725_OG-GT61-G_ANACO,((LOC103704408_OG-GT61-G_PHODA,(LOC105041546_modified_OG-GT61-G_ELAGV,(LOC105041545_OG-GT61-G_ELAGV,LOC105036721_OG-GT61-G_ELAGV)0.8140)0.7390)0.9960,LOC109829135_OG-GT61-G_ASPOF)0.4470)0.9970)0.5870,((LOC18593455_OG-GT61-G_THECC,LOC104877597_OG-GT61-G_VITVI)0.9670,(Cc05_g14110_OG-GT61-G_COFCA,Cc05_g14100_OG-GT61-G_COFCA)1.0000)0.9260)0.9860,((LOC109840057_OG-GT61-D4_ASPOF,(((Aco002125_OG-GT61-D4_ANACO,((LOC100836135_OG-GT61-D4_BRADI,LOC101784684_OG-GT61-D4_SETIT)0.8920,LOC4338684_OG-GT61-D4_ORYSA)1.0000)0.9630,Ma11_g18560_OG-GT61-D4_MUSAC)0.5070,(LOC103716439_OG-GT61-D4_PHODA,LOC105057566_OG-GT61-D4_ELAGV)0.9980)0.9220)0.9970,((LOC18426991_OG-GT61-F_AMBTC,((((LOC100241096_OG-GT61-F_VITVI,LOC18610312_OG-GT61-F_THECC)0.3920,LOC18610311_OG-GT61-F_THECC)0.8860,(AT2G03370_OG-GT61-F_ARATH,AT2G03360_OG-GT61-F_ARATH)1.0000)1.0000,LOC18426988_modified_OG-GT61-F_AMBTC)0.8670)0.9970,(LOC18442774_OG-GT61-D_AMBTC,((((Cc00_g29990_GT61-D_COFCA,(AT3G57380_OG-GT61-D_ARATH,AT2G41640_OG-GT61-D_ARATH)0.9890)0.0250,(AT3G10320_OG-GT61-D_ARATH,(LOC18598895_OG-GT61-D_THECC,LOC100245312_OG-GT61-D_VITVI)0.5860)0.2350)0.7450,Cc06_g17740_OG-GT61-D_COFCA)0.9010,(((((LOC100826343_OG-GT61-D2_BRADI,LOC4324540_OG-GT61-D2_ORYSA)0.8000,LOC101754751_OG-GT61-D2_SETIT)1.0000,Aco007260_OG-GT61-D2_ANACO)0.2250,LOC109831329_OG-GT61-D2-D3_ASPOF)0.5670,(((LOC103716931_OG-GT61-D3_PHODA,LOC105049132_OG-GT61-D3_ELAGV)1.0000,Aco019877_OG-GT61-D3_ANACO)0.8810,((LOC103719214_OG-GT61-D2_PHODA,LOC105045572_OG-GT61-D2_ELAGV)0.9970,(((LOC101759818_OG-GT61-D3_SETIT,LOC100829143_OG-GT61-D3_BRADI)0.6360,LOC4344156_OG-GT61-D3_ORYSA)1.0000,((Ma05_g18360_OG-GT61-D1_MUSAC,(Aco002317_OG-GT61-D1_ANACO,(LOC105049168_OG-GT61-D1_ELAGV,LOC109843905_OG-GT61-D1_ASPOF)0.9260)0.0760)0.8710,(LOC101758405_OG-GT61-D1_SETIT,(LOC101781379_OG-GT61-D1_SETIT,(LOC100826127_OG-GT61-D1_BRADI,LOC4325410_OG-GT61-D1_ORYSA)0.9600)0.7810)1.0000)1.0000)0.2140)0.0000)0.8460)0.9560)0.9360)0.2440)0.5940)1.0000)0.9420);

Groups G, D and F, 55 sequences (307 aligned positions).

(((((((((AT3G10320_OG-GT61-D_ARATH:0.21712700,(AT3G57380_OG-GT61-D_ARATH:0.12706100,AT2G41640_OG-GT61-D_ARATH:0.07619300)0.9970:0.10241600)0.4410:0.02696800,(LOC100245312_OG-GT61-D_VITVI:0.14157400,LOC18598895_OG-GT61-D_THECC:0.12228000)0.2530:0.02035000)0.6370:0.01764600,(Cc06_g17740_OG-GT61-D_COFCA:0.16202300,Cc00_g29990_OG-GT61-D_COFCA:0.23633500)0.7970:0.04509000)0.9450:0.04461000,LOC18442774_OG-GT61-D_AMBTC:0.27345900)0.7680:0.01528700,((((LOC103719214_OG-GT61-D2_PHODA:0.02288700,LOC105045572_OG-GT61-D2_ELAGV:0.03965000)0.9910:0.06354600,(((LOC100826343_OG-GT61-D2_BRADI:0.09079800,LOC4324540_OG-GT61-D2_ORYSA:0.03015700)0.1890:0.01390100,LOC101754751_OG-GT61-D2_SETIT:0.04142200)1.0000:0.13803100,Aco007260_OG-GT61-D2_ANACO:0.13452100)0.9390:0.05438900)0.4330:0.03590600,((LOC103716931_OG-GT61-D3_PHODA:0.05203300,LOC105049132_OG-GT61-D3_ELAGV:0.04743400)0.9980:0.10789500,(((LOC101759818_OG-GT61-D3_SETIT:0.02108300,LOC4324540_OG-GT61-D2_ORYSA:0.05391700)0.2190:0.02566800,LOC100829143_OG-GT61-D3_BRADI:0.09773600)1.0000:0.21435500,Aco019877_OG-GT61-D3_ANACO:0.06740700)0.9860:0.07805300)0.4590:0.02278200)0.8840:0.03119500,LOC109831329*_OG-GT61-D2-D3_ASPOF:0.17185100)0.9450:0.05068800)0.8670:0.04633000,(LOC109840057_OG-GT61-D4_ASPOF:0.16688200,((((LOC4338684_OG-GT61-D4_ORYSA:0.01222600,(LOC101784684_OG-GT61-D4_SETIT:0.01409900,LOC100836135_OG-GT61-D4_BRADI:0.03580000)0.8890:0.02003700)1.0000:0.23931200,Aco002125_OG-GT61-D4_ANACO:0.29100900)0.8840:0.09539400,Ma11_g18560_OG-GT61-D4_MUSAC:0.29108400)0.5330:0.05915600,(LOC103716439_OG-GT61-D4_PHODA:0.04519400,LOC105057566_OG-GT61-D4_ELAGV:0.03013200)0.9980:0.14144800)0.9110:0.06123800)1.0000:0.33039900)0.5990:0.02747500,(LOC109843905_OG-GT61-D1_ASPOF:0.27203500,(LOC105049168_OG-GT61-D1_ELAGV:0.19295400,((Aco002317_OG-GT61-D1_ANACO:0.21304000,(LOC101781379_OG-GT61-D1_SETIT:0.21259900,((LOC101758405_OG-GT61-D1_SETIT:0.12801900,LOC4325410_OG-GT61-D1_ORYSA:0.13668300)0.0660:0.04702900,LOC100826127_OG-GT61-D1_BRADI:0.26744900)0.3190:0.02649400)1.0000:0.45870800)0.9030:0.06932800,Ma05_g18360_OG-GT61-D1_MUSAC:0.27413900)0.7590:0.02102500)0.8490:0.08826700)1.0000:0.22994200)0.8020:0.13926800,(((((LOC18610311_OG-GT61-F_THECC:0.24783700,LOC18610312_OG-GT61-FTHECC:0.28176700)0.3790:0.03798200,LOC100241096_OG-GT61-F_VITVI:0.32704700)0.9430:0.08611800,(AT2G03370_OG-GT61-F_ARATH:0.09083500,AT2G03360_OG-GT61-F_ARATH:0.06540000)1.0000:0.31776900)0.9990:0.19056100,LOC18426988_modified_OG-GT61-F_AMBTC:0.43611100)0.8160:0.05489500,LOC18426991_OG-GT61-F_AMBTC:0.40010700)0.9870:0.19308600)1.0000:0.19646417,((LOC18433656_OG-GT61-G_AMBTC:0.38503300,(LOC109829135_OG-GT61-G_ASPOF:0.25747800,((LOC103704408_OG-GT61-G_PHODA:0.05294800,(LOC105041546_modified_OG-GT61-G_ELAGV:0.02870300,(LOC105041545_OG-GT61-G_ELAGV:0.00350100,LOC105036721_OG-GT61-G_ELAGV:0.00347400)0.9040:0.01421600)0.7560:0.01078300)1.0000:0.20025900,Aco024725_OG-GT61-G_ANACO:0.24383500)0.2200:0.07647400)0.9980:0.19284700)0.1120:0.06078700,((LOC18593455_OG-GT61-G_THECC:0.17300600,LOC104877597_OG-GT61-G_VITVI:0.32465400)0.9390:0.10974300,(Cc05_g14110_OG-GT61-G_COFCA:0.00000100,Cc05_g14100_OG-GT61-G_COFCA:0.00000100)1.0000:0.45132600)0.9730:0.14999600)1.0000:0.31941383);

Group A, 164 sequences (277 aligned positions).

(((LOC18433845_OG-GT61-A_AMBTC:0.38265600,LOC18433847_OG-GT61-A_AMBTC:0.31802900)0.4380:0.04688000,(((LOC100261238_OG-GT61-A_VITVI:0.18551000,(LOC18604238_OG-GT61-A_THECC:0.24947400,Cc04_g06720_OG-GT61-A_COFCA:0.23980700)0.9770:0.10113100)0.7260:0.04073400,(Cc04_g06730_OG-GT61-A_COFCA:0.34614500,((LOC18604233_OG-GT61-A_THECC:0.09642000,LOC18604231_OG-GT61-A_THECC:0.17279500)0.9200:0.05946400,(AT3G18180_OG-GT61-A_ARATH:0.10646600,AT3G18170_OG-GT61-A_ARATH:0.03907400)1.0000:0.28365900)0.9460:0.08166700)0.2000:0.03787400)0.9810:0.09582900,(((LOC101764052_OG-GT61-A6_SETIT:0.21100400,((LOC4340861_OG-GT61-A6_ORYSA:0.24616100,LOC100832680_OG-GT61-A6_BRADI:0.18540900)0.7930:0.06445800,(LOC101767467_OG-GT61-A6_SETIT:0.07058400,LOC101765418_OG-GT61-A6_SETIT:0.12465600)0.9730:0.09738800)0.2750:0.03799500)1.0000:0.34185200,((LOC109829083_OG-GT61-A6_ASPOF:0.21574100,(Aco023932_OG-GT61-A6_ANACO:0.14839600,Aco012670_OG-GT61-A6_ANACO:0.22884200)0.7700:0.05629500)0.1440:0.00852300,(((LOC103696248_OG-GT61-A6_PHODA:0.04493000,LOC105041147_OG-GT61-A6_ELAGV:0.03722500)0.9960:0.09382800,(Ma07_g11810_OG-GT61-A6_MUSAC:0.12144600,(Ma04_g30870_OG-GT61-A6_MUSAC:0.08584800,Ma02_g16320_OG-GT61-A6_MUSAC:0.05804400)0.9760:0.06515300)1.0000:0.20020200)0.7510:0.03083000,(Ma08_g03850_OG-GT61-A6_MUSAC:0.21154500,Ma01_g16460_OG-GT61-A6_MUSAC:0.13641000)0.9120:0.05676500)0.9000:0.03117500)0.3990:0.04281900)0.9980:0.15332000,((((Aco021831_pseudo_OG-GT61-A2_ANACO:0.26842600,((((LOC4351859_OG-GT61-A2_ORYSA:0.11762700,LOC101783129_OG-GT61-A2_SETIT:0.07723100)0.3250:0.01906800,LOC101761394_OG-GT61-A2_SETIT:0.02540800)0.9670:0.09615100,((LOC101783535_OG-GT61-A2_SETIT:0.09209100,LOC4326077_OG-GT61-A2_ORYSA:0.11812500)0.2910:0.02482900,LOC100843951_OG-GT61-A2_BRADI:0.12111400)0.8130:0.06313800)1.0000:0.40252300,((((LOC101783666_OG-GT61-A2_SETIT:0.05899800,LOC100845100_OG-GT61-A2_BRADI:0.05362500)0.8880:0.01807700,LOC4329205_OsXAT2_OG-GT61-A2_ORYSA:0.04009700)0.9940:0.07423100,((LOC100837177_OG-GT61-A2_BRADI:0.05224300,LOC4333275_OsXAT3_OG-GT61-A2_ORYSA:0.01835500)0.1250:0.00758800,LOC101779994_OG-GT61-A2_SETIT:0.03258400)0.9710:0.05019900)0.9950:0.09987500,Aco012681_OG-GT61-A2_ANACO:0.09067500)0.9650:0.07382800)0.8660:0.04562000)0.9070:0.07711100,((((Ma10_g25290_OG-GT61-A2_MUSAC:0.18485500,Ma07_g11840_OG-GT61-A2_MUSAC:0.10044600)0.8870:0.03651300,(Ma04_g30890_OG-GT61-A2_MUSAC:0.06870700,Ma02_g16300_OG-GT61-A2_MUSAC:0.15285300)0.8960:0.03106600)0.9700:0.05483000,(Ma02_g24680_OG-GT61-A2_MUSAC:0.09607600,Ma10_g20100_OG-GT61-A2_MUSAC:0.11803500)0.9990:0.11392300)0.9340:0.04895400,(((LOC103719019_OG-GT61-A2_PHODA:0.06696900,LOC105048468_OG-GT61-A2_ELAGV:0.02407900)0.9320:0.05766900,(LOC103696198_OG-GT61-A2_PHODA:0.04646200,LOC105041136_OG-GT61-A2_ELAGV:0.05379800)0.9020:0.03546400)0.9990:0.12261100,(LOC103696188_OG-GT61-A2_PHODA:0.06261500,LOC105041135_OG-GT61-A2_ELAGV:0.09700200)1.0000:0.17842900)0.8750:0.03884200)0.3840:0.04788400)0.9880:0.08232900,(LOC103705636_OG-GT61-A?_PHODA:0.07175900,LOC105049280_OG-GT61-A?_ELAGV:0.06408700)0.9990:0.21384000)0.8210:0.02589200,((((Aco010901_OG-GT61-A5_ANACO:0.13913300,(LOC101780012_OG-GT61-A5_SETIT:0.15870500,LOC100846082_OG-GT61-A5_BRADI:0.15043700)1.0000:0.29673300)0.9400:0.05971200,((((Ma07_g11830_OG-GT61-A5_MUSAC:0.33448500,(Ma09_g00650_OG-GT61-A5_MUSAC:0.08642800,Ma01_g14200_OG-GT61-A5_MUSAC:0.07001500)0.9980:0.17350800)0.9630:0.09712200,(Ma04_g30880_OG-GT61-A5_MUSAC:0.03834900,Ma02_g16310_OG-GT61-A5_MUSAC:0.05061000)1.0000:0.22045900)0.9710:0.09731400,Ma02_g09010_OG-GT61-A5_MUSAC:0.30618800)0.6870:0.03812500,(LOC103697726_OG-GT61-A5_PHODA:0.05287800,LOC105041557_OG-GT61-A5_ELAGV:0.03562300)0.9970:0.10265000)0.7670:0.02725800)0.9160:0.04279800,LOC109844158_OG-GT61-A5_ASPOF:0.28521300)0.9740:0.06605500,((((((((Ma09_g00680_OG-GT61-A3_MUSAC:0.08309200,(Ma01_g14160_OG-GT61-A3_MUSAC:0.07583200,Ma11_g12050_OG-GT61-A3_MUSAC:0.11232700)0.8320:0.03348900)0.9970:0.15133800,Ma11_g12040_OG-GT61-A3_MUSAC:0.22180300)0.9870:0.12459400,Ma06_g11090_OG-GT61-A3_MUSAC:0.26730300)0.2840:0.04408000,((Ma08_g16530_OG-GT61-A3_MUSAC:0.07370900,((Ma09_g00670_OG-GT61-A3_MUSAC:0.05799200,Ma01_g14180_OG-GT61-A3_MUSAC:0.04769900)0.2990:0.01630800,Ma01_g14170_OG-GT61-A3_MUSAC:0.09930400)0.4930:0.02248800)0.9580:0.07194400,Ma10_g20090_OG-GT61-A3_MUSAC:0.12188000)1.0000:0.20089600)0.8720:0.04675800,((((LOC4326083_OG-GT61-A3_ORYSA:0.05813400,(LOC101780825_OG-GT61-A3_SETIT:0.06511000,LOC101763272_OG-GT61-A3_SETIT:0.16305600)0.8260:0.03749600)0.6530:0.04527400,LOC100828889_OG-GT61-A3_BRADI:0.12083100)1.0000:0.27670400,((Aco021837_part2_OG-GT61-A3_ANACO:0.08159800,Aco021835_part2_OG-GT61-A3_ANACO:0.15439800)0.1000:0.02479800,Aco021835_part1_OG-GT61-A3_ANACO:0.05031000)0.9990:0.15534600)0.9730:0.09263600,(partial_OG-GT61-A3_PHODA:0.07586500,LOC105041137_OG-GT61-A3_ELAGV:0.05882300)0.9570:0.07631800)0.8800:0.04043100)0.7310:0.01974100,LOC109846408_OG-GT61-A3-A4_ASPOF:0.32767800)0.6860:0.02105700,(((LOC101782705_OG-GT61-A7_SETIT:0.12584000,LOC4326078_OG-GT61-A7_ORYSA:0.17065300)0.9960:0.11863200,Aco021833_part2_OG-GT61-A7_ANACO:0.25725000)0.1590:0.03036600,((((LOC101780400_OG-GT61-A7_SETIT:0.05323800,LOC9267972_OG-GT61-A7_ORYSA:0.04038800)0.5900:0.02506800,LOC100833936_OG-GT61-A7_BRADI:0.04903400)0.9810:0.07073600,(LOC101760861_OG-GT61-A7_SETIT:0.09428700,(LOC4329203_OG-GT61-A7_ORYSA:0.05780800,(LOC100825423_OG-GT61-A7_BRADI:0.04880100,LOC100844505_OG-GT61-A7_BRADI:0.04017600)0.9450:0.03415300)0.6910:0.02177900)0.9840:0.08042200)1.0000:0.15195900,Aco012680_OG-GT61-A7_ANACO:0.13359600)0.6550:0.05049400)1.0000:0.33662800)0.8000:0.02389800,((((LOC103696231_OG-GT61-A4_PHODA:0.03805300,LOC105041138_OG-GT61-A4_ELAGV:0.09395700)0.9990:0.15315500,(Aco021838_OG-GT61-A4_ANACO:0.00570800,Aco021837_part1_OG-GT61-A4_ANACO:0.03390500)1.0000:0.24050500)0.0000:0.01834600,((Ma09_g00660_OG-GT61-A4_MUSAC:0.07390300,Ma01_g14190_OG-GT61-A4_MUSAC:0.14208900)1.0000:0.24462300,((Ma03_g01160_OG-GT61-_MUSAC:0.14850400,Ma03_g21790_OG-GT61-A4_MUSAC:0.10572700)0.4630:0.02912300,Ma08_g16780_OG-GT61-A4_MUSAC:0.08323400)0.9910:0.09807400)0.9700:0.07154100)0.4290:0.01084100,(((LOC105041144_OG-GT61-A4_ELAGV:0.06397600,(LOC105041141_OG-GT61-A4_ELAGV:0.03416400,LOC105041139_OG-GT61-A4_ELAGV:0.05800200)1.0000:0.10306400)0.8250:0.01304200,((LOC105041145_OG-GT61-A4_ELAGV:0.07979700,LOC103696210_OG-GT61-A4_PHODA:0.06582200)0.9360:0.03210400,(LOC105041142_OG-GT61-A4_ELAGV:0.04118700,LOC105041140_OG-GT61-A4_ELAGV:0.05840400)0.7900:0.02838300)0.7870:0.01565100)1.0000:0.12590100,((LOC4342010_OG-GT61-A4_ORYSA:0.03430000,(LOC101760298_OG-GT61-A4_SETIT:0.12088400,LOC100826010_OG-GT61-A4_BRADI:0.10624900)0.6840:0.02900900)1.0000:0.46397400,((Aco021833_part1_OG-GT61-A4_ANACO:0.09996100,Aco012679_OG-GT61-A4_ANACO:0.11760800)0.9850:0.08883700,(((LOC4335139_OG-GT61-A4_ORYSA:0.40497700,(((LOC101781902_OG-GT61-A4_SETIT:0.10677000,LOC4326082_OG-GT61-A4_ORYSA:0.13794600)0.2160:0.03374700,LOC100844253_OG-GT61-A4_BRADI:0.15647800)0.7890:0.04340800,((LOC101761786_OG-GT61-A4_SETIT:0.13927100,LOC101762600_OG-GT61-A4_SETIT:0.46203100)0.7870:0.05578100,LOC101762197_OG-GT61-A4_SETIT:0.23508300)0.4390:0.05493100)0.9970:0.16963000)0.0710:0.05202600,((LOC101782301_OG-GT61-A4_SETIT:0.04251300,LOC100840722_OG-GT61-A4_BRADI:0.23651900)0.8730:0.06796500,LOC4326079_OG-GT61-A4_ORYSA:0.09428500)1.0000:0.25512000)0.9660:0.09816700,(((Aco012676_OG-GT61-A4_ANACO:0.02158600,((Aco031505_OG-GT61-A4_ANACO:0.00000100,Aco012675_OG-GT61-A4_ANACO:0.00359700)0.9380:0.02999800,Aco012671_OG-GT61-A4_ANACO:0.04692600)1.0000:0.15047200)0.8780:0.02403900,(Aco012677_modified_OG-GT61-A4_ANACO:0.04091600,Aco012673_OG-GT61-A4_ANACO:0.08075000)0.9720:0.04521800)0.9970:0.13074500,((LOC101766267_OG-GT61-A4_SETIT:0.10277200,LOC4329195_OG-GT61-A4_ORYSA:0.06085300)1.0000:0.26396500,(((LOC100840829_OG-GT61-A4_BRADI:0.13057400,LOC4341034_OG-GT61-A4_ORYSA:0.05464500)0.7480:0.02717100,LOC101769202_OG-GT61-A4_SETIT:0.06398500)0.9880:0.15748800,(LOC101785420_OG-GT61-A4_SETIT:0.12039000,LOC101780003_OG-GT61-A4_SETIT:0.07774400)1.0000:0.33953800)0.9950:0.16618400)0.2320:0.03130000)0.9910:0.15199600)0.8570:0.05336900)0.9730:0.09523000)0.9410:0.07766100)0.6900:0.01916100)0.9560:0.03618500)0.8780:0.06070300)0.3780:0.02003000)0.9430:0.06450500)0.8830:0.04147700)0.9290:0.07156200)0.5870:0.02966915,((LOC109843948_OG-GT61-A1_ASPOF:0.51915700,LOC109844962_OG-GT61-A1_ASPOF:0.36651100)0.9740:0.17796000,(((Os02g0135500_OG-GT61-A1_ORYSA:0.09255200,LOC100835033_OG-GT61-A1_BRADI:0.12259000)0.9450:0.04695500,(LOC4342012_OG-GT61-A1_ORYSA:0.04556300,LOC100826633_OG-GT61-A1_BRADI:0.07479900)1.0000:0.18611900)1.0000:0.14905300,((Aco010900_modified_OG-GT61-A1_ANACO:0.09303200,(((((LOC100833330_OG-GT61-A1_BRADI:0.05363400,Os10g0492200_OG-GT61-A1_ORYSA:0.04673500)0.1820:0.01371000,LOC101779596_OG-GT61-A1_SETIT:0.05041600)1.0000:0.11251600,((LOC101762945_OG-GT61-A1_SETIT:0.04929900,Os02g0331200_OG-GT61-A1_ORYSA:0.03842600)0.4640:0.01705200,LOC100838010_OG-GT61-A1_BRADI:0.08473200)0.6760:0.02815200)0.9920:0.08268500,Aco012682_modified_OG-GT61-A1_ANACO:0.11344300)0.9130:0.03703600,Aco021830_OG-GT61-A1_ANACO:0.19793900)0.8340:0.02704500)0.9340:0.05320100,(((LOC103719015_OG-GT61-A1_PHODA:0.05999000,LOC105048469_OG-GT61-A1_ELAGV:0.06003800)0.8060:0.02120500,(LOC103696170_OG-GT61-A1_PHODA:0.03672700,LOC105041134_OG-GT61-A1_ELAGV:0.04569700)0.9860:0.06174700)0.9850:0.07879100,((Ma01_g14150_OG-GT61-A1_MUSAC:0.04959700,Ma11_g12060_OG-GT61-A1_MUSAC:0.10268800)0.9970:0.09434200,((Ma06_g11080_OG-GT61-A1_MUSAC:0.12458900,Ma10_g20110_OG-GT61-A1_MUSAC:0.09547000)0.9280:0.03151300,Ma02_g24690_OG-GT61-A1_MUSAC:0.04329800)0.9390:0.04190600)0.9960:0.08545000)0.9150:0.05475200)0.8430:0.07328500)0.9580:0.12238900)0.5870:0.05748685);

PAML tree, 103 sequences (311 aligned positions)

((((((((((((LOC103696231_OG-GT61-A4_PHODA:0.05170900,LOC105041138_OG-GT61-A4_ELAGV:0.10706000)0.9980:0.14537700,(Aco021838_OG-GT61-A4_ANACO:0.00661700,Aco021837_part1_OG-GT61-A4_ANACO:0.04453800)1.0000:0.31155500)0.0270:0.02651300,(((LOC103696210_OG-GT61-A4_PHODA:0.07055700,LOC105041145_OG-GT61-A4_ELAGV:0.08879000)0.9380:0.03088300,(LOC105041144_OG-GT61-A4_ELAGV:0.07399200,(LOC105041142_OG-GT61-A4_ELAGV:0.05364900,LOC105041140_OG-GT61-A4_ELAGV:0.04888200)0.1360:0.01540400)0.2890:0.02755900)0.1580:0.01379000,(LOC105041141_OG-GT61-A4_ELAGV:0.03849700,LOC105041139_OG-GT61-A4_ELAGV:0.06576900)0.9990:0.09692300)1.0000:0.14164400)0.1260:0.02430700,(((Ma03_g01160_OG-GT61-A4_MUSAC:0.15246500,Ma03_g21790_OG-GT61-A4_MUSAC:0.12388600)0.7150:0.03071900,Ma08_g16780_OG-GT61-A4_MUSAC:0.08815200)0.9940:0.10511100,(Ma09_g00660_OG-GT61-A4_MUSAC:0.08767600,Ma01_g14190_OG-GT61-A4_MUSAC:0.16380200)1.0000:0.26060500)0.9760:0.06963400)0.2080:0.00982700,(((((Aco031505_OG-GT61-A4_ANACO:0.00000100,Aco012675_OG-GT61-A4_ANACO:0.00343000)0.9550:0.03627300,Aco012671_OG-GT61-A4_ANACO:0.04819100)1.0000:0.14834900,Aco012676_OG-GT61-A4_ANACO:0.04254100)0.8480:0.02868400,(Aco012677_modified_OG-GT61-A4_ANACO:0.04808600,Aco012673_OG-GT61-A4_ANACO:0.08586400)0.9040:0.04438300)1.0000:0.34426900,(Aco021833_part1_OG-GT61-A4_ANACO:0.13425100,Aco012679_OG-GT61-A4_ANACO:0.12971100)0.9820:0.10017900)1.0000:0.18482600)0.9230:0.03548600,((((partial_OG-GT61-A3_PHODA:0.07837600,LOC105041137_OG-GT61-A3_ELAGV:0.05496100)0.8860:0.07890500,((Aco021837_part2_OG-GT61-A3_ANACO:0.08304100,Aco021835_part2_OG-GT61-A3_ANACO:0.17190100)0.1190:0.02571600,Aco021835_part1_OG-GT61-A3_ANACO:0.06536000)1.0000:0.26433300)0.8030:0.05718700,(((((Ma11_g12050_OG-GT61-A3_MUSAC:0.13810900,Ma01_g14160_OG-GT61-A3_MUSAC:0.08968000)0.3090:0.02845500,Ma09_g00680_OG-GT61-A3_MUSAC:0.11276800)0.9990:0.16303200,Ma11_g12040_OG-GT61-A3_MUSAC:0.24980200)0.9830:0.12940400,Ma06_g11090_OG-GT61-A3_MUSAC:0.31300600)0.6150:0.05516500,((((Ma09_g00670_OG-GT61-A3_MUSAC:0.06291200,Ma01_g14180_OG-GT61-A3_MUSAC:0.05487400)0.6850:0.02115500,Ma01_g14170_OG-GT61-A3_MUSAC:0.10804600)0.7910:0.03421700,Ma08_g16530_OG-GT61-A3_MUSAC:0.06924200)0.9470:0.07406100,Ma10_g20090_OG-GT61-A3_MUSAC:0.14627400)1.0000:0.23645600)0.4730:0.05357600)0.2290:0.02793700,(Aco021833_partl2_OG-GT61-A3_ANACO:0.27095500,Aco012680_OG-GT61-A3_ANACO:0.19482100)1.0000:0.37135500)0.9050:0.04213200)0.9530:0.06448300,((LOC105049280_OG-GT61-A_ELAGV:0.31895400,(((Aco021831_pseudo_OG-GT61-A2_ANACO:0.30461300,Aco012681_OG-GT61-A2_ANACO:0.20951200)0.9850:0.10909500,(((Ma07_g11840_OG-GT61-A2_MUSAC:0.11292300,Ma10_g25290_OG-GT61-A2_MUSAC:0.18571600)0.9480:0.05180300,(Ma04_g30890_OG-GT61-A2_MUSAC:0.07239000,Ma02_g16300_OG-GT61-A2_MUSAC:0.17235400)0.9090:0.03285900)0.9710:0.06436300,(Ma02_g24680_OG-GT61-A2_MUSAC:0.10368200,Ma10_g20100_OG-GT61-A2_MUSAC:0.12687100)1.0000:0.12370600)0.8530:0.04384100)0.2980:0.04343300,(((LOC103719019_OG-GT61-A2_PHODA:0.08866400,LOC105048468_OG-GT61-A2_ELAGV:0.02439700)0.9530:0.05830200,(LOC103696198_OG-GT61-A2_PHODA:0.05446300,LOC105041136_OG-GT61-A2_ELAGV:0.05880700)0.9350:0.04384200)1.0000:0.15698400,(LOC103696188_OG-GT61-A2_PHODA:0.08175100,LOC105041135_OG-GT61-A2_ELAGV:0.10281700)1.0000:0.20046700)0.2820:0.02434700)0.9940:0.10044500)0.1190:0.02713300,((((((Ma09_g00650_OG-GT61-A5_MUSAC:0.10692600,Ma01_g14200_OG-GT61-A5_MUSAC:0.06314400)0.9980:0.17040300,Ma07_g11830_OG-GT61-A5_MUSAC:0.34693700)0.9690:0.10197600,(Ma04_g30880_OG-GT61-A5_MUSAC:0.04061900,Ma02_g16310_OG-GT61-A5_MUSAC:0.06145100)1.0000:0.21657600)0.9610:0.09396400,Ma02_g09010_OG-GT61-A5_MUSAC:0.33015500)0.2220:0.04235800,(LOC103697726_OG-GT61-A5_PHODA:0.05352500,LOC105041557_OG-GT61-A5_ELAGV:0.03680400)1.0000:0.13528000)0.5670:0.04928900,Aco010901_OG-GT61-A5_ANACO:0.21543600)0.9920:0.10806400)0.8150:0.01570200)0.8930:0.07107000,((((LOC103696248_OG-GT61-A6_PHODA:0.04105600,LOC105041147_OG-GT61-A6_ELAGV:0.03549200)1.0000:0.10801400,(Ma07_g11810_OG-GT61-A6_MUSAC:0.14309700,(Ma04_g30870_OG-GT61-A6_MUSAC:0.09355400,Ma02_g16320_OG-GT61-A6_MUSAC:0.06495400)0.9780:0.06371900)1.0000:0.21355200)0.9670:0.05461900,(Ma08_g03850_OG-GT61-A6_MUSAC:0.24677100,Ma01_g16460_OG-GT61-A6_MUSAC:0.15647700)0.8950:0.04513600)0.2870:0.00795100,(Aco023932_OG-GT61-A6_ANACO:0.14737700,Aco012670_OG-GT61-A6_ANACO:0.29963500)0.9780:0.06811600)1.0000:0.25778100)0.8780:0.03045000,((Cc04_g06720_OG-GT61-A_COFCA:0.36302500,(LOC100261238_OG-GT61-A_VITVI:0.22911800,((LOC18604233_OG-GT61-A_THECC:0.12003200,LOC18604231_OG-GT61-A_THECC:0.16321700)0.8380:0.06042700,(AT3G18180_OG-GT61-A_ARATH:0.10335500,AT3G18170_OG-GT61-A_ARATH:0.05607500)1.0000:0.30889100)0.9840:0.10906600)0.6410:0.03748200)0.7480:0.05086400,Cc04_g06730_OG-GT61-A_COFCA:0.39028600)0.9710:0.09378100)0.9290:0.05643500,(LOC18433847_OG-GT61-A_AMBTC:0.38195600,LOC18433845_OG-GT61-A_AMBTC:0.46137900)0.5200:0.05219300)0.6200:0.03907600,((((LOC103719015_OG-GT61-A1_PHODA:0.06168400,LOC105048469_OG-GT61-A1_ELAGV:0.06286200)0.9330:0.03963900,(LOC103696170_OG-GT61-A1_PHODA:0.03133300,LOC105041134_OG-GT61-A1_ELAGV:0.04935400)0.9800:0.05234300)0.9930:0.08843100,((Aco021830_OG-GT61-A1_ANACO:0.22307200,Aco012682_modified_OG-GT61-A1_ANACO:0.16269200)0.3340:0.04281000,Aco010900_Modified_OG-GT61-A1_ANACO:0.11200700)0.9890:0.10928700)0.7220:0.04779700,(((Ma06_g11080_OG-GT61-A1_MUSAC:0.13802800,Ma10_g20110_OG-GT61-A1_MUSAC:0.11079400)0.9140:0.03456000,Ma02_g24690_OG-GT61-A1_MUSAC:0.04747400)0.9150:0.04021500,(Ma01_g14150_OG-GT61-A1_MUSAC:0.05554800,Ma11_g12060_OG-GT61-A1_MUSAC:0.11001900)1.0000:0.11140100)0.9520:0.06693300)0.9990:0.26917700)1.0000:0.13263305,((LOC18433656_OG-GT61-G_AMBTC:0.34393300,((LOC103704408_OG-GT61-G_PHODA:0.06291600,(LOC105041545_OG-GT61-G_ELAGV:0.00517800,LOC105036721_OG-GT61-G_ELAGV:0.00523700)0.8360:0.02313800)1.0000:0.25270500,Aco024725_OG-GT61-G_ANACO:0.19728400)0.9990:0.29277700)0.4380:0.07071400,(LOC18593455_OG-GT61-G_THECC:0.26596700,(Cc05_g14110_OG-GT61-G_COFCA:0.00000100,Cc05_g14100_OG-GT61-G_COFCA:0.00000100)1.0000:0.44754300)0.9690:0.16910100)1.0000:0.18229595);
